# Probiotic *Limosilactobacillus Reuteri* (*Lactobacillus Reuteri*) Extends the Lifespan of *Drosophila Melanogaster* through Insulin/IGF-1 Signaling

**DOI:** 10.14336/AD.2023.0122

**Published:** 2023-08-01

**Authors:** Hye-Yeon Lee, Ji-Hyeon Lee, Seung Hyung Kim, Su-Yeon Jo, Kyung-Jin Min

**Affiliations:** ^1^Department of Biological Sciences and Bioengineering, Inha University, Incheon 22212, Korea; ^2^Institute of Traditional Medicine and Bioscience, Daejeon University, Daejeon 34520, Korea; ^3^WEDEA Co., Science Park 305, HNU, Daejeon 34054, Korea

**Keywords:** Probiotics, *Limosilactobacillus reuteri*, Lifespan, *Drosophila melanogaster*, Reuterin, Insulin/IGF-1 signaling

## Abstract

The term probiotic refers to bacteria that provide a beneficial effect to the host. *Limosilactobacillus reuteri* (*Lactobacillus reuteri*) is a probiotic isolated from human breast milk. Although *L. reuteri* has antimicrobial and anti-inflammatory activities occasionally linked to anti-aging effects, there are no reports of the effects of *L. reuteri* on longevity. This study evaluated the anti-aging effects of *L. reuteri* on the lifespan and physiology of *Drosophila melanogaster*. *L. reuteri* increased the mean lifespan of fruit flies significantly without reducing the reproductive output, food intake, or locomotor activity. Furthermore, the data suggested that the longevity effect of *L. reuteri* is mediated by the reduction of the insulin/IGF-1 signaling pathway and the action of reuterin, an antimicrobial compound produced by *L. reuteri*. These results show that *L. reuteri* can be used as a probiotic that acts as a dietary restriction mimetic with anti-aging effects.

## INTRODUCTION

The average life expectancy of humans has increased with advances in medical technology and improved quality of life. Accordingly, many studies examined ways to live a long and healthy life and delay aging. To this end, people’s interest in dietary supplements for health and anti-aging effects is increasing. Probiotics are health-functional foods that have attracted attention in recent years. The World Health Organization defines probiotics as “live microorganisms that confer a health benefit to the host when administered in adequate amounts”. Examples include *Bifidobacterium* and *Lactobacilli* [1]. Several studies have examined the effect of probiotics on the host physiology, including the attenuation of metabolic syndrome [2], prevention of oxidative stress-related diseases [3], improvement in intestinal dysbiosis [4], modulation of the immune function [5], and lifespan extension [6].

This study examined the longevity effect of *Limosilactobacillus reuteri* (*Lactobacillus reuteri*), a bacteria found in human breast milk [7]. Previous studies have shown that *L. reuteri* is an effective probiotic with a preventive effect on hypercholesterolemia [8], an antimicrobial effect against pathogens [9], and an anti-inflammatory effect [10]. Several studies in rodents have also shown that *L. reuteri* could prevent obesity and diabetes and improve the health and physiology of organisms [7, 11-13]. In particular, *L. reuteri* reduces the body weight, fat content, and cholesterol in high-energy fed-induced obese rodent models [7, 12, 14]. However, there is no definitive evidence of a pro-longevity effect of *L. reuteri*, even though these effects, such as gut health improvement, obesity prevention, and immunity regulation, are related to longevity. Moreover, the identification of secondary metabolite influencing the host physiology and lifespan is necessary for *L. reuteri* - it is known that secondary metabolites produced from microorganisms, such as short-chain fatty acids, directly affect the host [15].

This study investigated the longevity effect, underlying mechanism, and the active compound of the probiotic *L. reuteri* in *Drosophila melanogaster*, a useful model animal for examining aging and microbe-host interactions. The results suggest that the probiotic *L. reuteri* extends the lifespan of *D. melanogaster* by reducing insulin/IGF-1 signaling (IIS). These results suggest that the probiotic *L. reuteri* may be an excellent candidate as an anti-aging supplement.

## MATERIALS AND METHODS

### Fly husbandry

All flies were cultured and reared at 25°C and 65% humidity on 12:12 hour light:dark cycles. Larval crowding was avoided by laying approximately 150 eggs on 250 cm^3^ fly bottles containing 25-30 mL of medium and were developed until the eclosion to adult. Standard cornmeal-sugar-yeast with agar (CSY) medium was used to rear the fly larvae, and standard sugar-yeast (SY) medium was provided when the flies were enclosed as adults. Table S10 lists the detailed recipes. Axenic (Ax) flies were generated by bleaching the embryos with a 5% sodium hypochlorite solution (Wako, Japan). The eggs under Ax conditions were passed through repeated generations and became third-generation flies. The Ax conditions were confirmed by plating a fly homogenate on the plate count agar (PCA, Neogen Corporation, MI, USA) and by 16S rRNA gene PCR using a bacterial 16S rRNA universal primer (27F and 1492R) provided by Macrogen (Seoul, South Korea).

### Supplementation of *L. reuteri* or reuterin

Freeze-dried *L. reuteri* powder was used in this study. For the fruit flies, dried *L. reuteri* was dissolved in distilled water and added to the SY food during food preparation at a final concentration of 50, 100, or 250 μg/mL. Reuterin was dissolved in distilled water and added to SY food during food preparation at final concentrations of 5, 50, or 500 μg/mL. For high-fat diet-induced obesity mice, 100 mg/kg *L*. *reuteri* was administered orally twice daily for 10 weeks. Dried *L. reuteri* was provided by Wedea Co. (Daejeon, Korea). After the inventors of Wedea Co. isolated only lactic acid bacteria from a woman's breast milk using a lactic acid bacteria selective medium, they selected a strain having acid, bile, and antibiotic resistance. The *L. reuteri* strain was isolated and identified by performing a molecular phylogenetic taxonomic analysis based on the 16S rRNA nucleotide sequence for the selected strain. Reuterin was purchased from Angene International Limited (Nanjing, China).

### Lifespan assays

**Figure 1.**
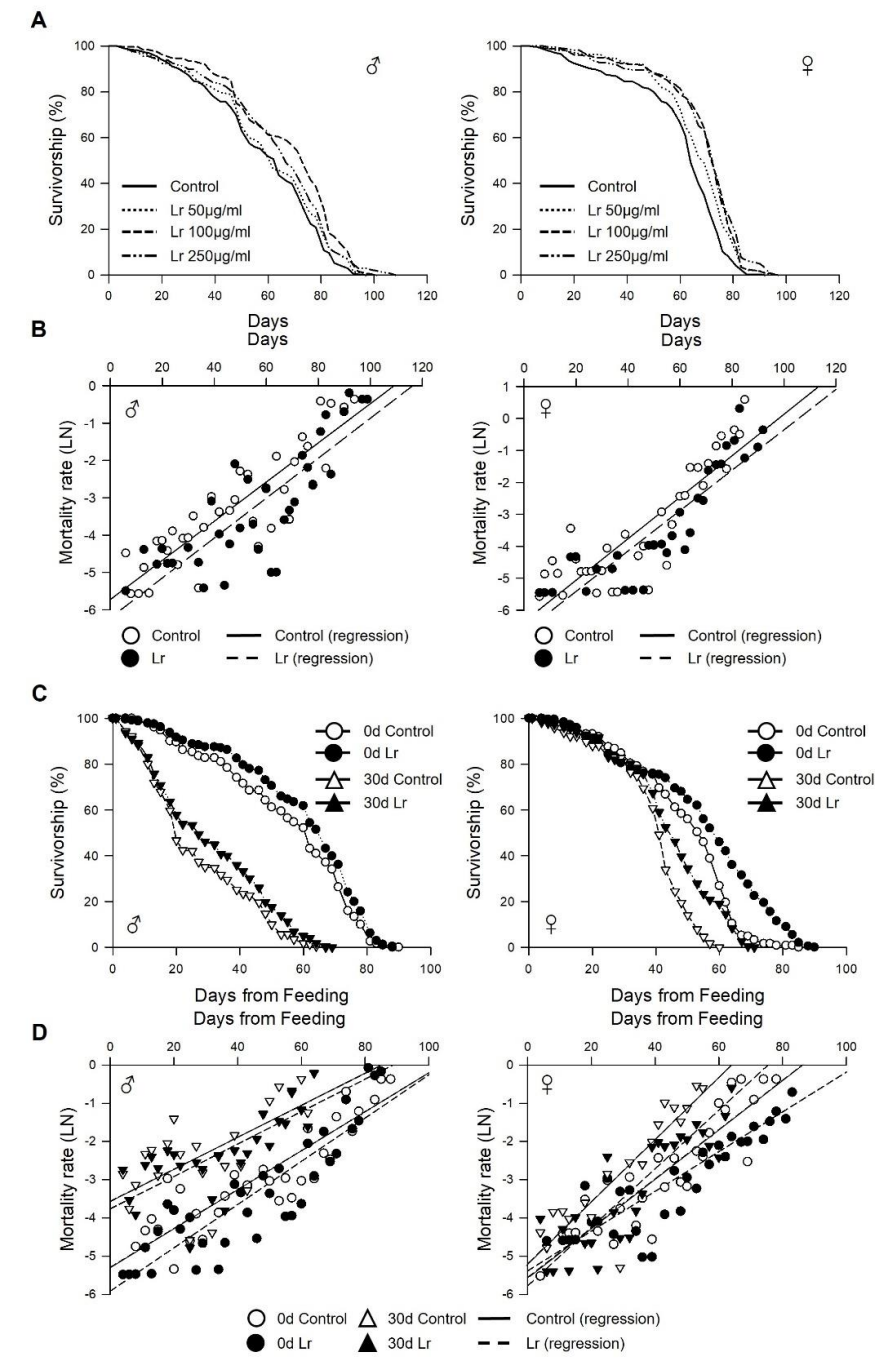
**Effect of *Limosilactobacillus reuteri* on lifespan in fruit flies**. (**A**) Effect of *L. reuteri* supplementation on the lifespan of males (left panel) and females (right panel). (**B**) Mortality curves of male (left panel) or female (right panel) flies supplemented with control (open dots) or *L. reuteri* (closed dots). (**C**) Effect of *L. reuteri* supplementation on the lifespan of male (left panel) and female (right panel) flies fed *L. reuteri* from 30 days after eclosion. (**D**) Mortality curves for male (left panel) and female (right panel) flies supplemented with the control (open symbols) or *L. reuteri* (closed symbols). The circles indicate the survival of flies fed *L. reuteri* from day 0 after eclosion (0 d), and the triangles indicate the survival of flies fed *L. reuteri* from 30 days after eclosion (30 d). The solid line indicates the regression of control flies, and the dashed line indicates the regression of flies fed *L. reuteri*. The natural log of the mortality rate was plotted using the Gompertz mortality model.

For the conventional lifespan assay (Fig. 1 and Fig. 6), newly eclosed 100 male and 100 fertilized female adult fruit flies were transferred to a 500 cm^2^ demography cage using CO_2_ anesthesia. For the lifespan assay under DR conditions (Fig. 5), 100 two- to three-day-old males or fertilized females were housed separately in a demography cage. Three replicate cages were set up for each group. For the axenic lifespan assay (Fig. 8), 20 newly eclosed males were transferred to a vial with axenic sterile SY food using CO_2_ anesthesia after having a two-day mating time. Ten replicate vials were set up for each group. Vials containing fresh SY food with/without *L. reuteri* or reuterin were fixed to cages and changed every two days. All dead flies were removed and recorded. The Kaplan-Meier survival estimator was used to estimate the survival function from lifetime data. Log-rank tests were carried out to determine the statistical significance of the differences in the mean lifespan. The JMP statistical package (SAS, NC, USA) was used for the analyses. This lifespan experiment was performed independently twice, and the results were reproducible.

### Fecundity

Male and female fruit flies were collected separately every three hours after initiating the first eclosion. Female virginity was confirmed by the absence of progeny in the food after 24 hours. The vials were set up on day two with a population density of two males and one virgin female. Every 24 hours, the flies were transferred to new vials containing fresh SY food with/without *L. reuteri*. The daily number of eggs laid by each female was counted for 24 days. Twenty replicate vials were tested per treatment. The statistical probability was determined using the *t*-test or Wilcoxon rank sum test.

### Feeding amount

After feeding the *L. reuteri*-supplemented diet for two or five weeks, 10 single-sex flies were transferred to a new vial containing the same diet with blue-dye number 1 (0.05% wt./vol) added. After feeding for 10 min, the anesthetized flies were washed with phosphate-buffered saline (PBS) and homogenized in 0.2 mL distilled water after head removal. After centrifugation for 5 min at 13,000 r/min (8,400 × g), the absorbance of the supernatants was measured at 595 nm using a spectrophotometer (Sunrise, Tecan, Austria). Three replicate vials were tested per treatment. The statistical probability was determined using a *t*-test. This experiment was performed at least three times independently. Data are presented as mean ± standard error of the mean (SEM) values and statistical probability was determined using a *t*-test.

### Climbing ability

Adult fruit flies were fed SY food with/without *L. reuteri* for five weeks. Every week, 10 single-sex flies were loaded into the vertical climbing assay apparatus, which was then tapped on a tabletop three times in rapid succession to initiate the negative geotaxis responses in the flies. The positions of the flies in the tubes were captured by digital images taken four seconds after initiating the geotactic behavior, and the height of flies from bottom was determined. The flies were assessed in consecutive trials separated by one minute of rest. Twenty replicates were used in four experiments per treatment. All data follows a normality distribution, and statistical probability was determined using a *t*-test.

### Development

The eggs were collected for 12 hours, and samples containing 20 eggs were transferred to the *L. reuteri*-containing SY food. Twenty replicate vials were established per group. The numbers of newly transformed pupae and adults were counted every six hours. The data are presented as mean ± SEM values, and statistical probability was determined using the Wilcoxon rank sum test.

### Analysis of commensal microbes in the flies

For CFU determination, five males were rinsed in 70% ethanol for three seconds for surface decontamination and then homogenized in sterile distilled water. The homogenates were diluted as necessary and plated onto PCA media, MRS media, or *Acetobacter*-selective media (ASM). At least five replicates were established for each group. The data are presented as mean ± SEM values and statistical probability was determined using a *t*-test for PCA media groups or Wilcoxon rank sum test for MRS and AS media groups.

For 16S rRNA PCR, total genomic DNA from 50 guts of male flies was extracted by using a DNeasy Blood & Tissue Kit (#69506, Qiagen, Hilden, Germany) in accordance with the manufacturer’s instructions. The PCR assays were performed at a 60°C annealing temperature and for 40 cycles using universal primers (27F and 1492R) and taxon-specific 16S rRNA gene primers for *Lactobacillus* or *Acetobacter* designed using Primer3 software, as well as universal PCR primers.

To determine the dominant commensal bacteria species in the gut of flies, Illumina sequencing analysis of the 16S rRNA gene was performed. The 16S rRNA gene amplicons from 50 dissected guts (comprising the Malpighian tubules and the crop) of males were analyzed by pyrosequencing using the Illumina iSeq 100 instrument (Illumina, San Diego, US). Phylogenetic relationships were determined by using Ezbiocloud.

### Body weight

Newly eclosed adult flies were collected for two days. Ten flies were assigned under mild CO_2_ anesthesia and transferred randomly to a vial containing sterile SY food with an *L. reuteri* supplement. Fifteen replicate vials were established for each group. The vials were changed every three days for new vials containing fresh sterile food. After three and five weeks, the body weight of the flies was measured on a microbalance (PAG214C, OHAUS, USA) after CO_2_ anesthesia. Data are presented as mean ± SEM values, and statistical probability was determined using a *t*-test (Male, 0 and 5 weeks; Female, 0, 2, and 5 weeks) or Wilcoxon rank sum test (Male, 2 weeks).

### Triacylglycerol (TAG) levels

The TAG level was measured, as described previously [16]. Newly eclosed flies were pretreated with *L. reuteri* 100 μg/mL for five weeks. TAG reagent (T2449, Sigma-Aldrich) or 1×PBST (PBS + 0.1% Triton X-100) was added to the heat-treated homogenized samples from 20 flies with the heads removed to determine total free glycerol or free glycerol levels. After incubation at 37°C for 30 min, the absorbance of the samples was measured at 540 nm using a spectrophotometer (Sunrise, Tecan, Austria). Free glycerol reagent (F6428, Sigma-Aldrich, USA) was added to the samples in which the absorbance was measured. After incubating the samples for 5 min at 37°C, the absorbance of the samples was measured at 540 nm using a spectrophotometer to determine the glycerol concentration. The TAG concentration was determined by subtracting the free glycerol concentration in the untreated samples from the total glycerol concentration in samples incubated with the triglyceride reagent. Three biological replicates were tested per treatment. Briefly, 2.5 mg/ml triolein equivalent glycerol standard (G7793, Sigma-Aldrich, USA) was used to determine the glycerol concentration. The TAG level was normalized to the protein content because the water content can affect the body weight. The data are presented as mean ± SEM values, and statistical probability was determined using a Wilcoxon rank sum test.

### Water content

Newly eclosed flies were pretreated with 100 μg/mL *L. reuteri* for five weeks. Ten flies per sample were weighed, dried for 48 hours at 70°C, and then weighed again. The water content was determined from the difference in weight divided by the initial weight. Fifteen replicates per treatment were used. The data are presented as mean ± SEM values, and statistical probability was determined using the Wilcoxon rank sum test for males or a *t*-test for females.

### Starvation resistance test

After feeding with the *L. reuteri* supplement for seven days, 10 single-sex fruit flies were transferred to vials containing 0.8% agar. Dead flies were counted every three hours. Fifteen replicates were established. The Kaplan-Meier survival estimator was used to estimate the survival function from the lifetime data. Log-rank tests were conducted to determine the statistical significance of differences in the mean lifespan. The JMP statistical package (SAS, NC, USA) was used for the analyses.

### Western Blot Analysis and Quantification

After feeding the fruit flies with 100 μg/mL *L. reuteri* for five weeks, the total proteins from 50 male fly heads were lysed with a PRO-PREP protein extraction buffer (#17081, iNtRON Biotechnology, South Korea) and centrifuged for 10 minutes at 13,000 r/min. Three times were conducted independently, and two replicates per trial were tested. The supernatants of the samples were quantified using a Bradford assay, and an equal amount of protein was loaded. Western Blot was performed with rabbit polyclonal anti-*Drosophila* phospho-Ser505 AKT antibody (1:1000, #4054, Cell Signaling, USA) and rabbit monoclonal anti-*Drosophila* AKT antibody (1:1000, #9272, Cell Signaling, USA). Rabbit monoclonal anti-α tubulin antibody (1:10000, #SAB4500087, Sigma-Aldrich, USA) was used as the loading control. The goat anti-rabbit IgG-HPR (1:5000, #sc-2004, Santa Cruz, USA) was used as the secondary antibody. The phospho-Ser505 AKT/AKT ratio was quantified using ImageJ software. The data are presented as mean ± SEM values, and the statistical probability was determined using a *t*-test.

### Real-time quantitative PCR

After feeding the fruit flies with 100 μg/mL *L. reuteri* or 5 μg/mL reuterin for five weeks, the total RNA was extracted from 50 male fly heads for insulin-like peptide genes or 15 male fly bodies for FOXO-target genes using RNAiso (Takara Bio, Japan). The total RNA (2 μg) was reverse transcribed using M-MLV reverse transcriptase (Promega, WI, USA). Quantitative PCR was performed using the Prism 7500 Sequence Detection System (Applied Biosystem, CA, USA) and TOPreal^TM^ qPCR 2× PreMix (Enzynomics, South Korea) according to the manufacturer’s instructions. At least three replicates were established for each group, and all experiments were repeated at least three times. The data are presented as the mean ± standard error of the mean. *Ribosomal protein 49* (*rp49*) was used as the internal control. Supplementary Table 11 lists the primer oligonucleotide sequences. The data are presented as mean ± SEM values, and statistical probability was determined using a *t*-test or Wilcoxon rank sum test.

### Immunohistochemistry

Adult male flies supplemented with the *L. reuteri* supplement for five weeks were dissected, and the adult fat body attached to the abdominal cuticles was isolated in 4% formaldehyde (F8775, Sigma-Aldrich, USA). After fixation with 4% formaldehyde for 1 h and washing with PBST, the samples were incubated overnight with the primary antibodies in PBST-2% bovine serum albumin (BSA) at 4°C. The samples were then washed with PBST, incubated with the secondary antibodies for 60 minutes at 25°C, washed with PBST, and mounted with Vectashield mountant (Vector Labs, USA). Anti-dFOXO antibody (a gift from O. Puig) and Cy^TM^3-conjugated anti-rabbit IgG secondary antibody (#111-165-045, Jackson Immuno Research Laboratories, USA) were diluted to 1:500 and 1:300, respectively, in PBST-2%BSA solution. DAPI was used to counterstain the nuclei. The resulting samples were assessed using confocal laser-scanning microscopy (LSM510 META, Carl Zeiss, Germany). At least 10 male flies were conducted for this test, and the experiments were repeated three times. The number of localizations of FOXO in the nucleus is presented as mean ± SEM values. The statistical probability was determined using a *t*-test or Wilcoxon rank sum test.

### Anti-obesity effect of *L. reuteri* in mouse

*Husbandry*: All mice used in this study were the male C57BL/6J strain purchased from Samtako Bio (Osan, South Korea). All experimental procedures involving mice were approved by the Institutional Animal Care and Use Committee (IACUC) and conducted according to the National Institutes of Health guidelines. Seven-week-old mice, 24 g in weight, were housed in a 22 ± 2°C and 55 ± 15% humidity room on 12:12 hour light:dark cycles with free access to water, standard chow (Protein 18.1%; Fat 5.1%; Fiber 4.8%; Mineral and Vitamin 2.9%; Carbohydrate 65.2%; Moisture < 10%; Samyang Feed Production Co., Wonju, South Korea) or high-fat chow (HFD, 60 kcal% of energy from fat, Protein 26.2%; Fat 34.9%; Fiber 6.5%; Mineral and Vitamin 6.8%; Carbohydrate 25.6%; #D12492, Research Diets Inc, USA).

*Body weight*: The body weight and food intake of 10 mice per group were recorded weekly for seven weeks, including at the beginning and end of the experiment. The weight was recorded on an electronic scale (CAS 2.5D, Seoul, Korea) once a week. The total weight gain was calculated as final body weight (g) - initial body weight (g). Average weight gain per day was measured according to the total body weight gain (g) /day. The food efficiency ratio (FER) was calculated as the total weight gain/total food intake × 100. The statistical probability was determined using a *t*-test.

*Autopsy and detection*: After administering *L*. *reuteri* for 10 weeks, the animals were fasted for three hours before sacrifice, and blood samples were collected. The organ tissues (liver and adipose tissue) of each experimental animal were extracted immediately and weighed after blood collection.

*Adipose tissue weighing*: After administering *L*. *reuteri* for 10 weeks, the abdominal subcutaneous fat and abdominal fat around the epididymis (liver and epididymal adipose tissue), kidney adipose tissue, and visceral fat were extracted from each experimental animal, and the weight of adipose tissue was calculated. The statistical probability was determined using a *t*-test or Wilcoxon rank sum test.

*Serum parameters*: After administering *L*. *reuteri* for 10 weeks, blood was collected by cardiac puncture and centrifuged at 3,000 r/min, 4 °C for 15 minutes within 30 minutes after blood collection to conduct blood biochemical tests. ALT and AST, which are indicators of liver function, total cholesterol, HDL (high-density lipoprotein), LDL (low-density lipoprotein), triglyceride, free fatty acid, and blood glucose, were measured using an automatic biochemical analyzer (Hitachi-720, Hitachi Medical, Japan) according to manufacturer protocols. To analyze insulin and adiponectin, microwells were coated with each antibody, and serum was dispensed. After dispensing the antibody avidin-HRP conjugate and the TMB substrate, the absorbance was measured at a wavelength of 450 nm using an ELISA reader (SpectraMax 190 Microplate Reader, Molecular Devices, USA). The statistical probability was determined using the *t*-test or Wilcoxon rank sum test.

*Oral glucose tolerance test (OGTT)*: The mice were denied access to food for 14 hours before the OGTT (n = 4). Blood samples (~10 μL) were taken from the tail vein at the 30-min interval after glucose administration. The blood glucose levels were measured from blood obtained by puncturing the tail vein using a OneTouch Select Plus Blood Glucose Meter (Johnson & Johnson, USA). The area under the curve (AUC) was calculated using ImageJ software. The statistical probability was determined using the Wilcoxon rank sum test.

### Statistical analysis

Log-rank tests were carried out to determine the statistical significance of the survival analysis results. The JMP statistical package (SAS, NC, USA) was used for the analyses. The test for normality (Shapiro-Wilk test) and the statistical probabilities (F-test, *t*-test, and Wilcoxon rank sum test) of data in this study were performed using R 4.2.2 software.

## RESULTS

### Dietary supplementation of *L. reuteri* prolongs the lifespan of the fly

To investigate the effect *L. reuteri* on the fruit fly lifespan, the lifespan of wild-type strain Canton-S flies was measured over a concentration range of *L. reuteri* powder from 50 to 250 μg/mL. The mean lifespan of the flies fed the *L. reuteri*-supplemented diet was increased significantly compared to that of the non-supplemented flies (Fig. 1A). In males, the mean lifespan of flies fed 100 or 250 μg/mL *L. reuteri* was 67.13 ± 1.34 days (14% increase, log-rank test, χ^2^ = 27.17, *p* < 0.0001) or 63.91 ± 1.35 days (9% increase, log-rank test, χ^2^ = 10.12, *p* < 0.005), respectively, while that of the non-treated control flies was 58.87 ± 1.31 days (Fig. 1A left, Supplementary Table 1). In addition, the age-related mortality was markedly lower in the male flies of all ages fed *L. reuteri* (Fig. 1B left, Supplementary Table 1). In the female flies, all *L. reuteri*-treated groups showed a longer lifespan compared to the non-treated control group (0 μg/mL, 60.66 ± 1.13 days; 50 μg/mL, 66.22 ± 0.95 days, 9% increase, log-rank test, χ^2^ = 15.74, *p* < 0.0001; 100 μg/mL, 68.85 ± 1.05 days, 14% increase, log-rank test, χ^2^ = 42.63, *p* < 0.0001; 250 μg/mL, 69.20 ± 1.13 days, 14% increase, log-rank test, χ^2^ = 48.55, *p* < 0.0001) (Fig. 1A right, Supplementary Table 1). These results suggest that a dietary *L. reuteri* supplement extends the lifespan of fruit flies, and 100 μg/mL *L. reuteri* is the optimal concentration in the fruit fly. Accordingly, a concentration of 100 μg/mL *L. reuteri* was used in subsequent experiments.

Next, this study investigated whether the longevity effect of *L. reuteri* supplementation persists when the flies are treated with *L. reuteri* in later life (Fig. 1C and 1D, Supplementary Table 1). Interestingly, the survival of 30-day-old male and female flies was increased significantly by *L. reuteri* supplementation (male, 30 days, 0 μg/mL, 26.36 ± 1.00 days, 100 μg/mL, 30.74 ± 1.23 days, 17% increase, log-rank test, χ^2^ = 11.92, *p* < 0.005; female, 30 days, 0 μg/mL, 39.21 ± 0.81 days, 100 μg/mL, 44.6 ± 1.03 days, 14% increase, log-rank test, χ^2^ = 40.71, *p* < 0.0001). Overall, supplementation with 100 μg/mL *L. reuteri* increases the lifespan of flies regardless of their age at which they started ingestion.

**Figure 2.**
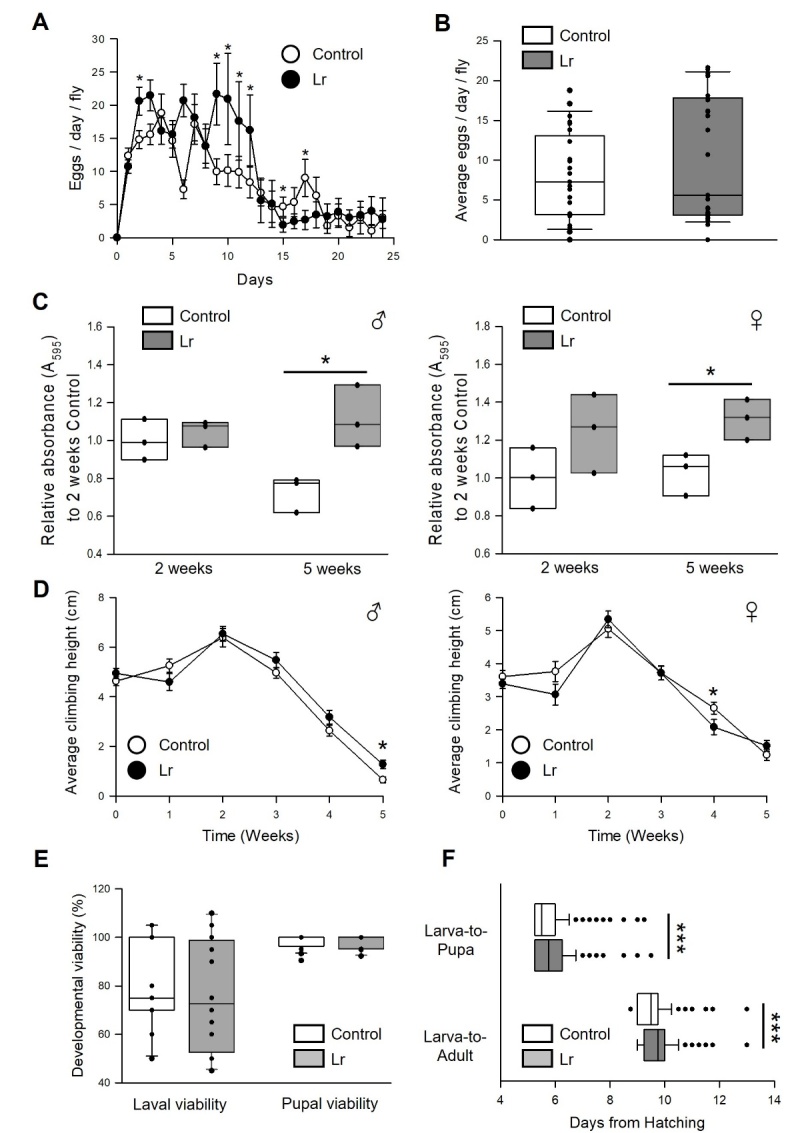
**Effect of *L. reuteri* on the physiology in fruit flies**. (**A**) Number of eggs per day per female for 25 days. The open circles indicate the number of eggs laid by flies fed control food, and the closed circles indicate the average number of eggs laid by flies fed *L. reuteri* (n = 20). Statistical probability was determined using a *t*-test (Day 1, 3-8, 13-16, 18-20) or Wilcoxon rank sum test (Day 2, 9-12, 17). (**B**) Average number of eggs laid by flies fed *L. reuteri* for 25 days. The white box indicates the average number of eggs laid by flies fed control food, and the grey box indicates the average number of eggs laid by flies fed *L. reuteri* (n = 24). Statistical probability was determined using the Wilcoxon rank sum test. (**C**) The effects of *L. reuteri* supplementation on the food intake in male (left panel) and female (right panel) flies fed *L. reuteri* for two or five weeks. The white bars indicate the food intake of flies fed control food and the black bars indicate the food intake of flies fed *L. reuteri* (n = 3). (**D**) The locomotor activity of male (left panel) or female (right panel) flies fed *L. reuteri* or a control food. The locomotor activity of flies was measured every week for five weeks. The open dots indicate the average climbing height of flies fed control food and the closed dots indicate the average climbing height of flies fed *L. reuteri* (n = 20). (**E**) The developmental viability of flies fed *L. reuteri* or a control food. Larval viability refers to the larva-to-pupa ratio, and pupal viability refers to the pupa-to-adult ratio. The white bars indicate the developmental viability of flies fed control food and the black bars indicate the developmental viability of flies fed *L. reuteri* (n = 20). The statistical probability was determined using the Wilcoxon rank sum test. (**F**) Developmental timing of flies fed *L. reuteri* or a control food. The white bars indicate the days from hatching of flies fed control food and the black bars indicate the days from hatching of flies fed *L. reuteri* (n > 300). The asterisks indicate significant differences compared to the control, * *p* < 0.05, *** *p* < 0.0001.

### Longevity effect of *L. reuteri* did not lead the adverse effects on the physiology of the fly

The physiological state, such as reproduction, food intake, mobility, developmental viability, and abundance of intestinal bacteria, is related to the lifespan of organisms [17, 18]. The fecundity, feeding rate, locomotor activity, developmental viability, and abundance of intestinal bacteria of flies with/without *L. reuteri* were measured to determine if the longevity effect of *L. reuteri* was caused by changes in the physiological states. For 25 days, both the number of eggs laid by each female fly per day and the average number of eggs were similar in the non-treated and *L. reuteri*-treated groups (Fig. 2A and 2B, 0 μg/mL, 8.45 ± 1.76; 100 μg/mL, 10.62 ± 2.58, *t*-test, *p* = 0.3). In addition, supplementation with *L. reuteri* did not reduce the food intake in the two-week-old flies (Fig. 2C, male, Wilcoxon rank sum test, *p* = 0.69; female, Wilcoxon rank sum test, *p* = 0.06), but increased the food intake for both sexes in five-week-old flies (Fig. 2C, male, 53% increase, Wilcoxon rank sum test, *p* < 0.05; female, 27% increase, Wilcoxon rank sum test, *p* < 0.05). In the locomotion test, *L. reuteri* supplementation did not change the locomotor activity of flies of both sexes (Fig. 2D) except for the five-week-old male flies, which showed increased locomotive activity (95% increase, *t*-test, *p* < 0.05). The administration of *L. reuteri* reduced the developmental timing without a change in developmental viability (Fig. 2E and 2F). Overall, these results suggest that the longevity effect of *L. reuteri* does not accompany the adverse effects on the physiological activity.

### Dietary supplementation of *L. reuteri* changed the microbial flora in flies

**Figure 3.**
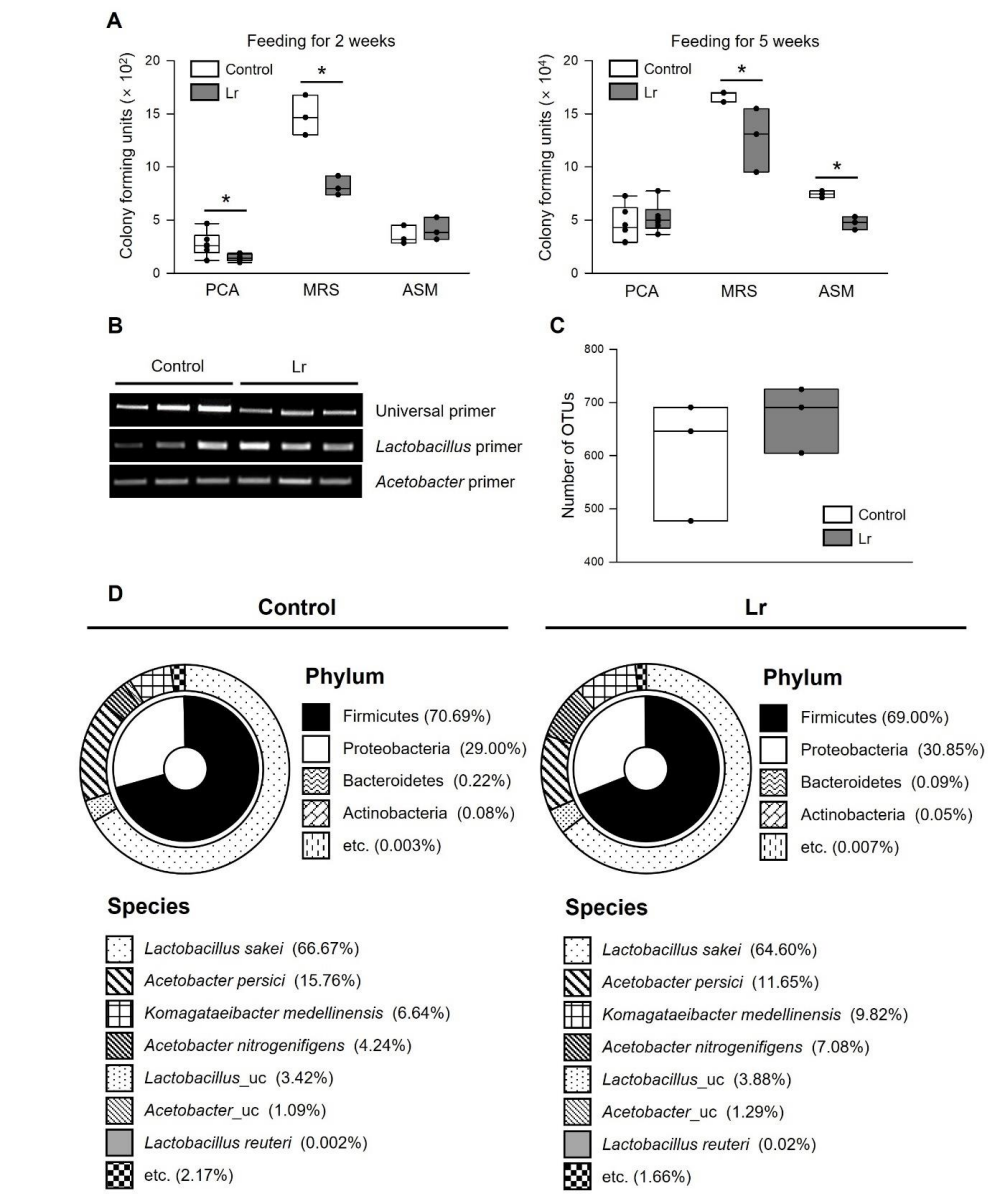
**Effect of *L. reuteri* on the commensal bacteria in fruit flies**. (**A**) Colony-forming units in flies fed *L. reuteri* or a control food. The white boxes indicate the CFU of flies fed control food, and the grey boxes indicate the CFU of flies fed *L. reuteri* (PCA, n = 6; MRS and ASM, n = 3). The asterisks indicate significant differences compared to the control, * *p* < 0.05. (**B**) PCR assay of microbial 16S rRNA amplified gene using universal (27F, 1492R), *Lactobacillus*-, or *Acetobacter*-specific primers. Microbial 16S rDNA gene sequences were amplified from genomic DNA extracted from the guts of three-week-old flies. (**C**) Assigned number of operational taxonomic units (OTUs) from Illumina sequencing data. (n = 3, Wilcoxon rank sum test, *p* = 0.38). (**D**) Double pie charts of three replicates of bacteria compositions of control or *L. reuteri*-fed fly guts. These charts show major phylum and species analyzed by Illumina sequencing of the 16S rRNA gene.

Treatment with probiotics positively alters the microbiome in the host [19]. Probiotic *L. reuteri* also affected the diversity and composition of gut microbiota in mice [20-24]. This study investigated how the *L. reuteri* treatment alters the microbiota in fruit flies because altered microbiota affects the host lifespan. Recent studies have shown that the increased abundance of commensal bacteria is closely related to health and aging in fruit flies [25, 26]. Therefore, this study examined the abundance of commensal bacteria in *L. reuteri*-fed flies using colony forming units test and 16S rRNA PCR. The abundance of commensal bacteria decreased significantly with *L. reuteri* supplementation (Fig. 3A and 3B universal primer), indicating that *L. reuteri* supplementation modulates the abundance of bacteria in flies. In the result of 16S rRNA PCR, supplementation of *L. reuteri* increased the load of *Lactobacillus*, but did not change the load of *Acetobacter*. However, by using Illumina sequencing analysis, *L. reuteri* supplementation did not significantly change the richness, diversity, and composition of commensal bacteria in the fruit fly’s gut (Fig. 3C-3D, Supplementary Table 2-3). Although this was not statistically significant, in control group, 604 operational taxonomic units (OTUs) were assigned; while, in the *L. reuteri* group, 673 OTUs were assigned (CD-HIT 97% threshold) (Fig. 3C and Supplementary Table 2), indicating that the compositional richness of the microbial species in the fruit fly’s gut slightly increased with *L. reuteri* supplementation. At the phylum level, Firmicutes (including *Lactobacillus*) and Proteobacteria (including *Acetobacter* and *Komagataeibacter*) comprised 99% of the microbiome in the *D. melanogaster* in this study (Fig. 3D and Supplementary Table 3). At the species level, *Lactobacillus sakei* and *Acetobacter persici* were dominant, and *Komagataeibacter medellinensis* and *Acetobacter nitrogenifigens* were slightly more detected than other bacteria in the gut of both groups of flies (Fig. 3D and Supplementary Table 3). For *L. reuteri*, the proportion of the total increased about 10 times in *L. reuteri*-fed fly compared to control suggesting that dietary supplementation of *L. reuteri* induces colonization of *L. reuteri* in the gut (Fig. 3D and Supplementary Table 3, Control, 0.002% of the whole bacteria; *L. reuteri*, 0.017% of the whole bacteria). These results indicate that the supplementation of *L. reuteri* affects the load of commensal bacteria in the fly gut, but not their diversity and composition.

**Figure 4.**
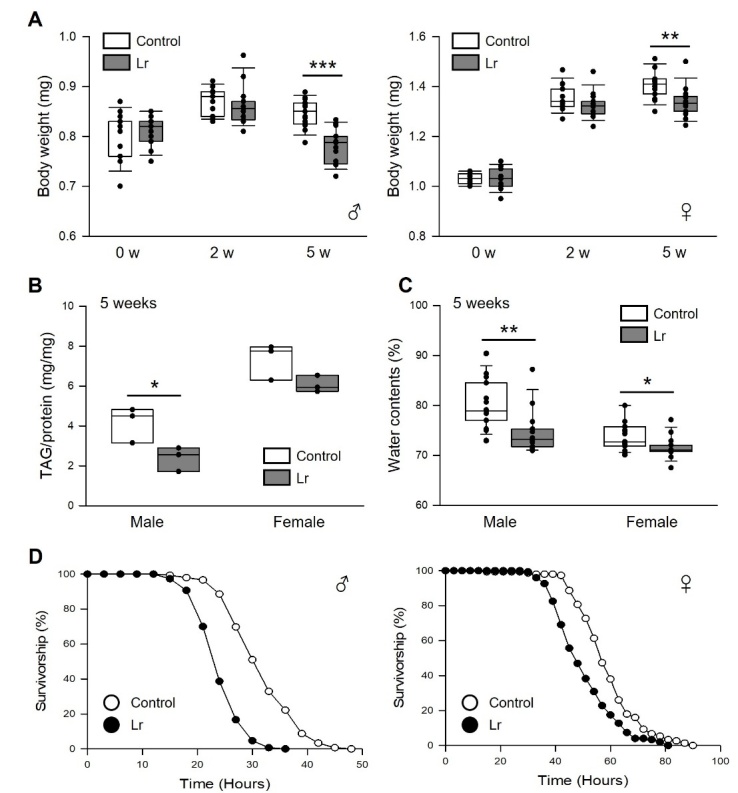
**The effect of *L. reuteri* on the body weight, TAG level, water contents, and starvation resistance of fruit flies**. (**A**) Body weight of male (left panel) or female (right panel) flies fed *L. reuteri* or control food. The white bars indicate the body weights of flies fed control food, and the black boxes indicate the body weights of flies fed *L. reuteri* (n = 15). The asterisks indicate significant differences compared to the control. (**B**) TAG contents of male and female flies fed *L. reuteri* or control food. The white bars indicate the TAG content of flies fed control food, and the black bars indicate the TAG content of flies fed *L. reuteri* (n = 3). (**C**) The water content of the flies fed a control diet (white bars) or an *L. reuteri*-containing diet (black bars) (n = 15). (**D**) Survival of male (left panel) or female (right panel) flies fed *L. reuteri* or control food for two weeks and then subjected to starvation stress. * *p* < 0.05, ** *p* < 0.005, *** *p* < 0.0001.

### Dietary supplementation of *L. reuteri* reduced the body weight, lipid contents, and water contents in flies

Supplementation with *L. reuteri* reduced the body weight and fat content, showing an anti-obesity effect in rodents and humans [11-13]. The fly body weight and amount of TAG, the primary lipid-storage molecule in insects, were measured to determine if the anti-obesity effect of *L. reuteri* is conserved in flies (Fig. 4A and 4B). Long-term supplementation with *L. reuteri* reduced the body weight of both sexes (Fig. 4A, male, five weeks, 8% decrease, *t*-test, *p* < 0.0001; female, five weeks, 5% decrease, *t*-test, *p* < 0.005). The body weight loss appears to be at least partly attributed to decreased TAG levels (Fig. 4B, male, 36% decrease, Wilcoxon rank sum test, *p* < 0.05; female, 6% decrease, Wilcoxon rank sum test, *p* = 0.06) and water contents in *L. reuteri*-treated flies (Fig. 4C, male, 7% decrease, Wilcoxon rank sum test, *p* < 0.005; female, 3% decrease, *t*-test, *p* < 0.05). The survival of flies following starvation stress was also measured because survival under starvation conditions is mainly affected by the amount of stored fat. Consistent with the observation, supplementation with *L. reuteri* markedly decreased the resistance to starvation stress in both sexes (Fig. 4D, male, 24% decrease, log-rank test, χ^2^ = 130.22, *p* < 0.0001; female, 14% decrease, log-rank test, χ^2^ = 28.32, *p* < 0.0001). Overall, supplementation with *L. reuteri* prevents weight gain and fat accumulation, even though food intake is increased in old flies (Fig. 2C).

### Longevity effect of *L. reuteri* is mediated through the IIS pathway

**Figure 5.**
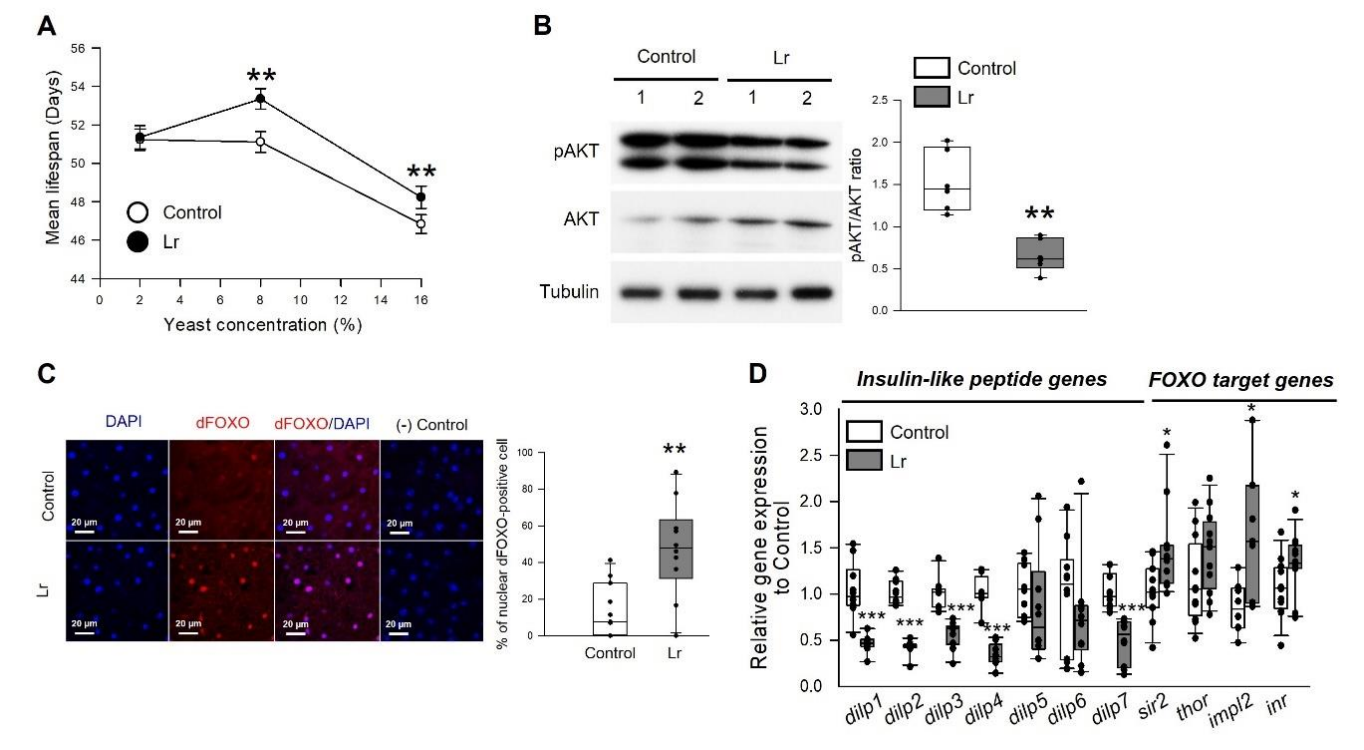
**Relationship between the longevity effect of *L. reuteri* and insulin signaling**. (**A**) Mean lifespan of flies fed 2, 8, or 16% yeast diet. The white circles indicate the mean lifespan of flies fed food without *L. reuteri* (Control), and the black circles indicate the mean lifespan of flies fed food with *L. reuteri* (Lr). The asterisks indicate significant differences compared to the control. (**B**) The level of phosphorylated AKT (pAKT)/AKT of flies fed a control diet or an *L. reuteri*-containing diet. The band density was analyzed with ImageJ, and the pAKT protein level was normalized to the total AKT (right, SEM, n = 6). (**C**) Translocalization of dFOXO to the nucleus following supplementation of the diet with *L. reuteri*. Negative controls represent the fat body tissue exposed to Cy3-conjugated secondary antibody only (no primary antibody) in addition to DAPI. The abdominal fat body was stained with anti-dFOXO (red) and DAPI (blue) (left). Percentage of the nuclear dFOXO-positive cells compared with cells stained with DAPI (right, SEM, control n = 11; Lr n = 10). The original magnification was 200×. The statistical probability was determined using the Wilcoxon rank sum test. (**D**) The mRNA levels of insulin-like peptide genes and FOXO-target genes were analyzed in the male fruit flies fed an *L. reuteri*-containing diet or a control diet for five weeks (IIS-related genes, n = 9; FOXO-target genes, n = 12). Statistical probability was determined using the Wilcoxon rank sum test (*dilp1-6*, *sir2*, and *impl2*) or *t*-test (*dilp7*, *thor*, and *inr*). * *p* < 0.05, ** *p* < 0.005, *** *p* < 0.0001.

Increased mobility (Fig. 2D), delayed developmental rate (Fig. 2F), decreased abundance of bacteria (Fig. 3A and 3B), and reduced body weight and fat accumulation (Fig. 4A and 4B) were observed, even though the feeding amounts to the flies were increased by supplementation with *L. reuteri* (Fig. 2C). Interestingly, all of these features were also observed in dietary restriction (DR). DR is a well-established strategy to extend the lifespan of an organism [17]. Several compounds have been reported to extend the lifespan of organisms via a mechanism similar to that of DR [27]. To determine if the longevity effect of *L. reuteri* is related to the DR mechanism, fruit flies were fed a 2, 8, or 16% yeast extract diet with/without the supplementation with *L. reuteri* (Fig. 5A and Supplementary Fig. 2). Male flies fed the 2 or 8% yeast diet had higher survival than male flies fed the 16% yeast diet (Supplementary Fig. 2, Supplementary Table 4, 16% yeast + control, 46.83 ± 0.49 days; 8% yeast + control, 51.11 ± 0.54 days, 9% increase, log-rank test, χ^2^ = 46.94, *p* < 0.0001; 2% yeast + control, 51.23 ± 0.56 days, 9% increase, log-rank test, χ^2^ = 41.91, *p* < 0.0001). *L. reuteri* supplementation significantly increased the mean and median lifespans of the flies fed the 8% and 16% yeast diets (Fig. 5A and Supplementary Fig. 2, Supplementary Table 4, control vs. Lr, 16% yeast + Lr, 48.23 ± 0.58 days, 3% increase, log-rank test, χ^2^ = 8.09, *p* < 0.005, 8% yeast + Lr, 53.35 ± 0.54 days, 4% increase, log-rank test, χ^2^ = 10.93, *p* < 0.005). On the other hand, the longevity effect of *L. reuteri* supplementation was abolished under the 2% yeast diet (Fig. 5A and Supplementary Fig. 2, Supplementary Table 4, control vs. Lr, 2% yeast + Lr, 51.36 ± 0.60 days, log-rank test, χ^2^ = 0.03, *p* = 0.87). These results suggest that *L. reuteri* can extend the lifespan of fruit flies in a similar manner to that of DR.

The longevity effect of DR is mediated by the inhibition of the IIS pathway and the activation of the forkhead box O (FOXO) transcription factor [28]. To determine if the *L. reuteri*-induced lifespan extension effect is associated with the IIS pathway, this study evaluated the ratio of phosphorylated AKT (pAKT) to the total AKT protein levels, the localization of dFOXO, and the expression of insulin-like peptides genes (Fig. 5B-5D). Compared to the non-feeding control group, the *L. reuteri*-fed group exhibited a significantly downregulated pAKT/AKT ratio (Fig. 5B, *t*-test, *p* < 0.005), and the dFOXO was translocated more clearly from the cytoplasm to the nucleus after *L. reuteri* supplementation for five weeks (Fig. 5C, Wilcoxon rank sum test, *p* < 0.005). Similarly, in *L. reuteri*-fed flies, the gene expression levels of *dilp1*, *dilp2*, *dilp3*, and *dilp4* decreased significantly, and the gene expression levels of *dilp5*, *dilp6*, and *dilp7* tended to decrease, but it was not statistically significant (Fig. 5D, Wilcoxon rank sum test or *t*-test). The expression levels of the target genes of the dFOXO transcription factor, such as *sir2*, *thor*, *impl2*, and *inr*, increased significantly in the flies fed *L. reuteri* for five weeks (Fig. 5D, Wilcoxon rank sum test or *t*-test). Thus, the life-extension effect of *L. reuteri* is related to the downregulation of the IIS pathway.

**Figure 6.**
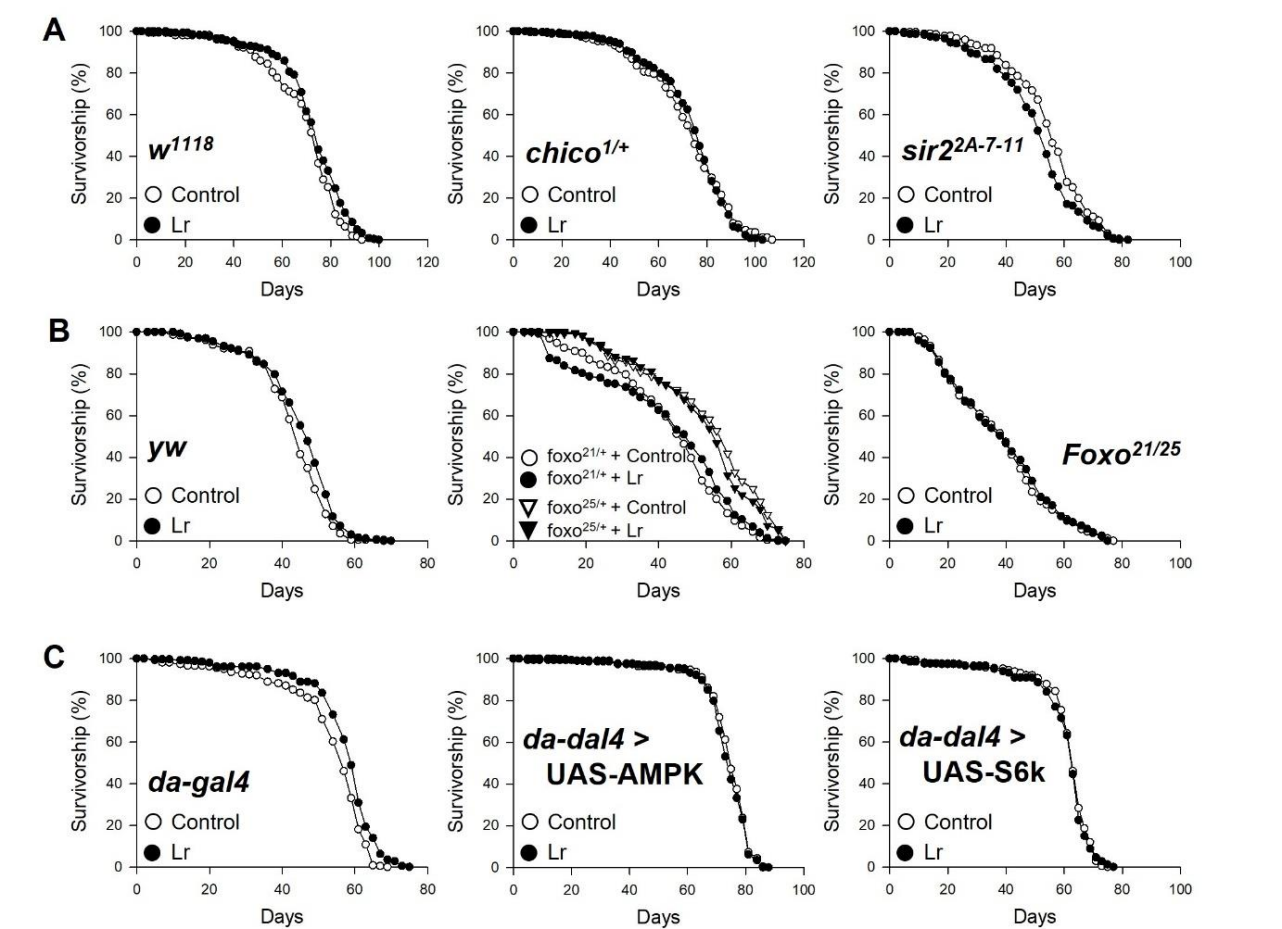
**Survival of mutant fly with *L. reuteri* supplementation**. (**A**) Survival of *w^1118^* (left), *chico* mutant (middle), and *dSir2* mutant (right). (**B**) Survival of *yw* (left), *foxo^21/+^* and *foxo^25/+^* mutant (middle), and *foxo^21/25^* mutant (right). (**C**) Survival of da-gal4 (left), da-gal4 > UAS-AMPK mutant (middle), and da-gal4 > UAS-S6K mutant (right). The white circles indicate the lifespan of the flies fed food without *L*. *reuteri* (Control), and the black circles indicate the lifespan of flies fed food with *L*. *reuteri* (Lr).

The lifespans of Insulin receptor substrate null mutant (*w^1118^;chico^1/+^*), sir2 null mutant (*w^1118^;sir2^2A-7-11^*), and dFOXO null mutant (*yw;foxo^21/25^*) flies were measured with/without *L. reuteri* supplementation to determine if the longevity effect of *L. reuteri* is mediated by IIS downregulation, or sirtuin activation, or both (Fig. 6A-6B and Supplementary Table 5). Like the results used wild-type Canton-S, wild-type control flies (*w^1118^* and *yw*), and da-gal4 control flies fed *L. reuteri* lived longer than the non-fed control flies (Fig. 6A left, *w^1118^*, 5% increase, log-rank test, χ^2^ = 12.34, *p* < 0.005; Fig. 6B left, *yw*, 4.5% increase, log-rank test, χ^2^ = 9.65, *p* < 0.005; Fig. 6C left, da-gal4, 7% increase, log-rank test, χ^2^ = 27.33, *p* < 0.001). On the other hand, the lifespans of *chico^1/+^*, *sir2^2A-7-11^*, and *foxo^21/25^* were not increased by *L. reuteri* supplementation (Fig. 6A middle, *chico^1/+^*, log-rank test, χ^2^ = 1.27, *p* = 0.26; Fig. 6A right, *sir2^2A-7-11^*, 8% decrease, log-rank test, χ^2^ = 10.36, *p* < 0.005; Fig. 6B right, *foxo^21/25^*, log-rank test χ^2^ = 0.17, *p* = 0.68), suggesting that IIS downregulation and sir2 activation mediate the longevity effect of *L. reuteri*. The AMPK (AMP-activated protein kinase) and dTOR (*Drosophila* target-of-rapamycin) pathways also represent the DR-related molecular mechanisms. The lifespans of the AMPK-overexpression mutant and S6K^KQ^ (a dominant-negative form of S6 kinase) mutant induced by da-gal4 were not increased by *L. reuteri* supplementation (Fig. 6C middle, da-gal4 > UAS-AMPK, log-rank test, χ^2^ = 1.17, *p* = 0.28; Fig. 6C right, da-gal4 > UAS- S6K^KQ^, log-rank test, χ^2^ = 0.35, *p* = 0.55). Overall, the mechanism of the life-extension effect of *L. reuteri* is closely related to the mechanism of the life-extension effect by dietary restriction.

**Figure 7.**
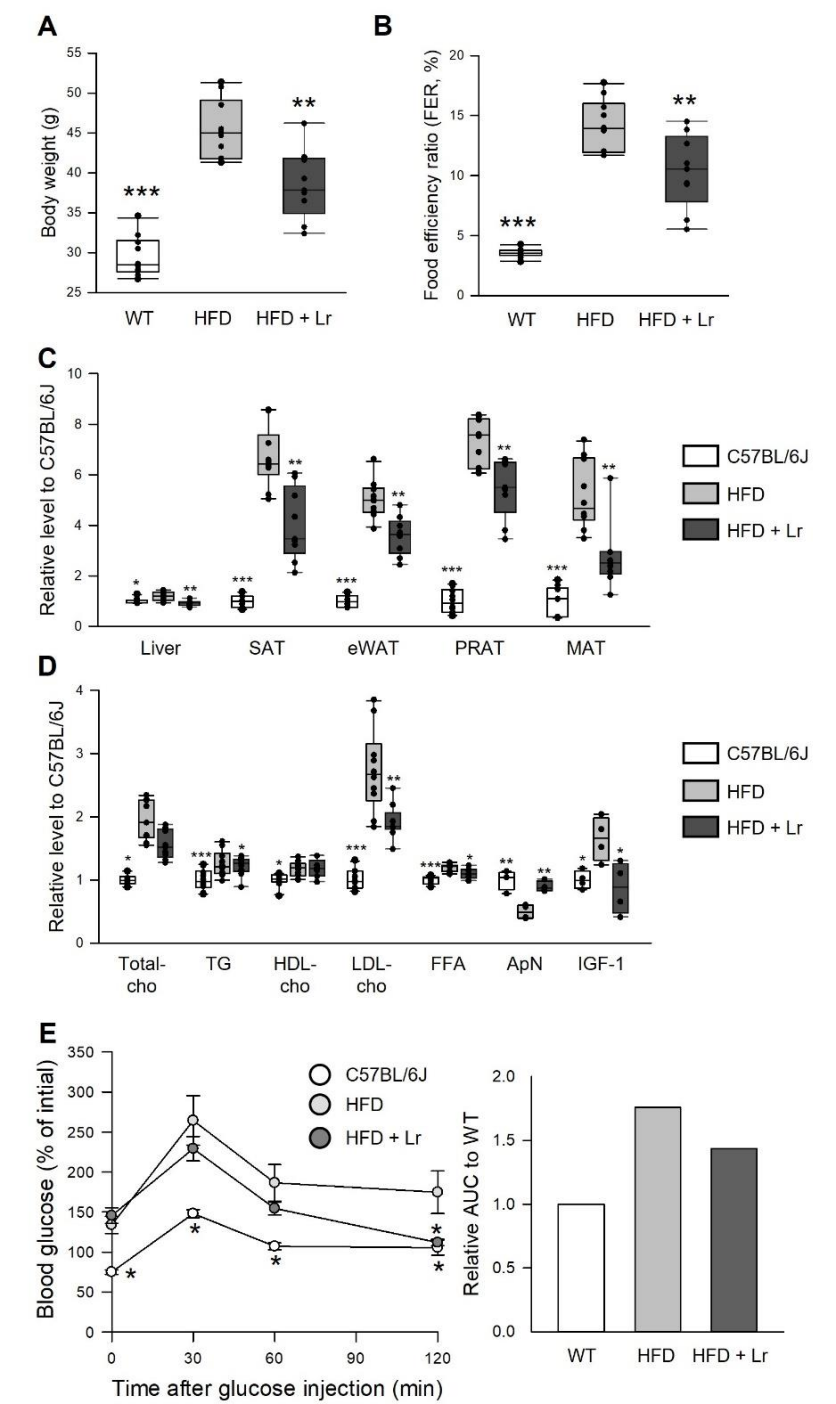
**Anti-obesity effect of *L***. *reuteri* in a high-fat diet-induced obesity mouse model. (A and B) Change in body weight (A) and food efficiency ratio (B) of mice fed a normal diet (WT), high-fat diet (HFD), or HFD with *L. reuteri* diet (HFD + Lr) for 7 weeks (n = 10). (C and D) Relative weight of the organ and fat (C) and relative level of lipid contents, adiponectin, and IGF-1 in serum (D) of mice fed a normal diet, HFD or HFD + Lr for seven weeks (lipid contents, n = 10; adiponectin and IGF-1, n = 4). Statistical probability was determined using the *t*-test (SAT, PRAT, Total-cho, TG, LDL-cho, FFA, ApN, and IGF-1) or Wilcoxon rank sum test (Liver, eWAT, MAT, and HDL-cho). (**E**) Blood glucose levels of mice after the oral glucose tolerance test (OGTT) (left) and the area under the curve (AUC) (right) of mice fed a normal diet, HFD, or HFD + Lr for 7 weeks (n = 5). The asterisks indicate significant differences compared to HFD control. * *p* < 0.05, ** *p* < 0.005, *** *p* < 0.0001. SAT, Abdominal subcutaneous adipose tissue; eWAT, Epididymal white adipose tissue; PRAT, Perirenal adipose tissue; MAT, Mesenteric adipose tissue; cho, Cholesterol; TG, Triglyceride; LDL, Low-density lipoprotein; HDL, High-density lipoprotein; FFA, Free fatty acid; ApN, Adiponectin; IGF-1, Insulin-like growth factor-1.

### Anti-obesity effect of *L. reuteri* in a high-fat diet-induced obesity mouse model

The above results showed *L*. *reuteri* have anti-obesity and insulin/IIS reduction effect and the lifespan extension effect of *L. reuteri* was more effective in a high-energy diet. The body weight, lipid contents, and glucose levels were tested in high-fat diet (HFD)-induced obesity C57BL/6J mice with *L*. *reuteri* supplementation to confirm that these anti-obesity and insulin reduction effects of *L*. *reuteri* are conserved to mammals under a high-energy diet (Fig. 7). The body weight of the mice was increased significantly after being treated with the HFD, but the supplementation of *L*. *reuteri* (HFD + Lr) decreased the body weight gain by HFD (Fig. 7A, *t*-test, *p* < 0.005). The food efficiency ratio showed the same pattern as the body weight change (Fig. 7B, *t*-test, *p* < 0.005). The HFD increased the liver weight, abdominal subcutaneous fat, and adipose tissues considerably. On the other hand, HFD + Lr reduced these weights significantly (Fig. 7C, Wilcoxon rank sum test or *t*-test, *p* < 0.005), suggesting that the body weight changes may be related to the weight changes in the liver, fat, and adipose tissues. Moreover, serum indicators and oral glucose tolerance were measured because biochemical parameter analysis in the serum and insulin/IGF-1 level was not performed in previous studies. Serum indicators, such as triglycerides, total cholesterol, high-density lipoprotein (HDL) cholesterol, low-density lipoprotein (LDL) cholesterol, and free fatty acid, were elevated in HFD mice, but supplementation of *L*. *reuteri* lowered these pathological indicators effectively (Fig. 7D and Supplementary Table 6). Furthermore, decreased adiponectin, an adipocyte-derived peptide with anti-inflammatory and insulin-sensitizing properties, and increased IGF-1 by the HFD were recovered by the supplementation of *L*. *reuteri* (Fig. 7D and Supplementary Table 6). Furthermore, the oral glucose tolerance test showed that *L*. *reuteri* effectively improved the decreased insulin sensitivity by the HFD (Fig. 7E). Overall, supplementation of *L*. *reuteri* had anti-obesity effects in the HFD-induced obesity mouse model, suggesting that the effect of *L*. *reuteri* observed in fruit flies are conserved in mammalian models.

### Reuterin may be a key active compound for the longevity effect of *L. reuteri*

This study used the freeze-dried powder of *L. reuteri*, which has no biological activity when ingested, indicating that secondary metabolite is responsible for the lifespan extension by *L. reuteri*. Reuterin is an antimicrobial compound produced by *L*. *reuteri*, but there are limited studies investigating the other effects of reuterin. Previous studies showed that a reuterin treatment inhibits the growth of pathogens, such as *Salmonella* spp., *Clostridioides difficile*, and *Helicobacter pylori* [29-31]. The results also showed that supplementation of *L. reuteri* decreased the abundance of intestinal bacteria (Fig. 3A and 3B). Because the previous study showed that the controlled abundance of bacteria increases the fly lifespan [26], it was hypothesized that reuterin acts as a secondary metabolite for the longevity effect of *L. reuteri*. This study first investigated whether the supplementation of reuterin decreased the abundance of intestinal bacteria in flies. Overall, reuterin inhibited the growth of bacteria extracted from fruit flies *in vitro* and reduced the abundance of bacteria in fruit flies *in vivo* (Supplementary Fig. 3).

**Figure 8.**
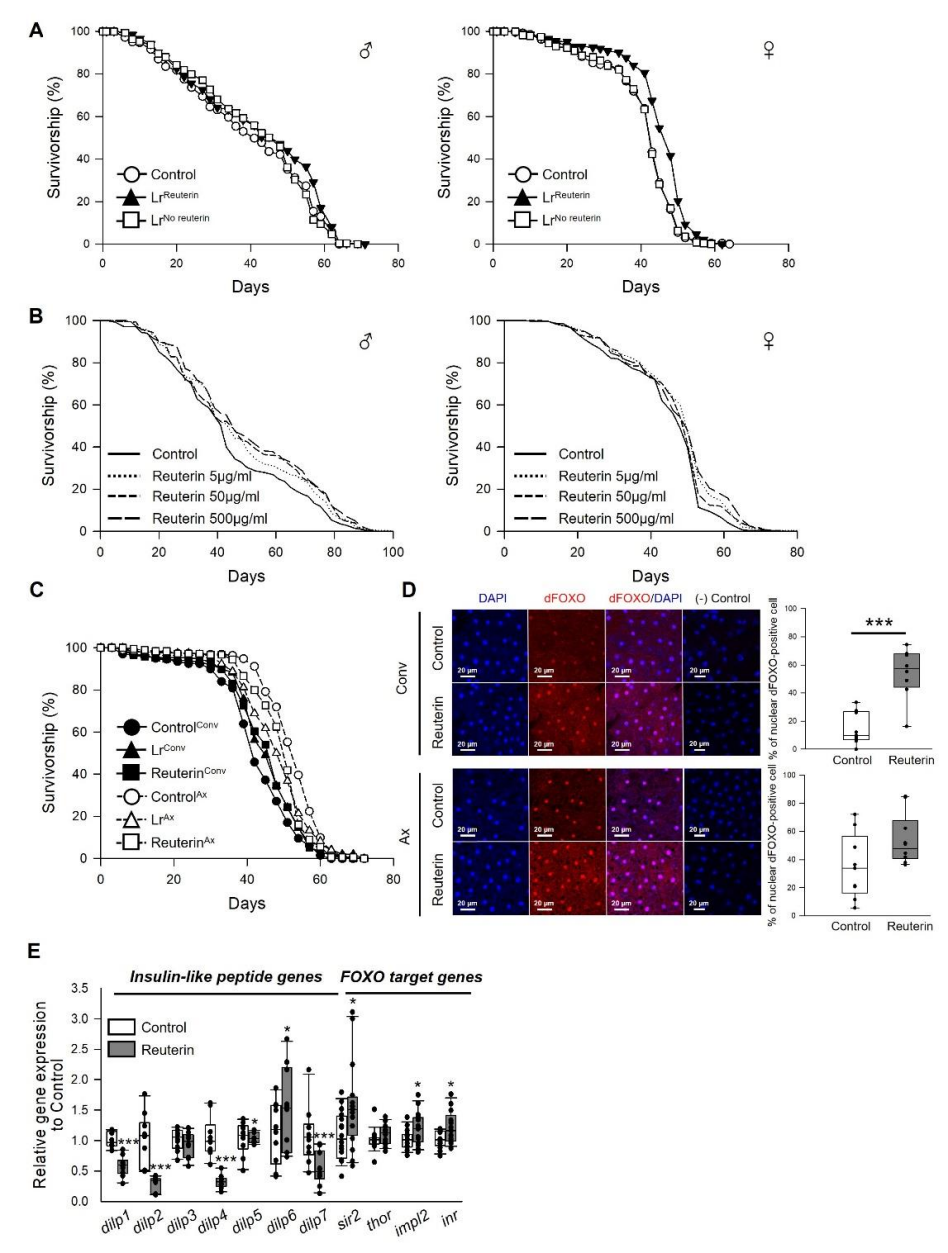
**Reuterin as a key compound of longevity effect of *L. reuteri***. (**A**) Lifespan of males (left panel) and females (right panel) fed reuterin-producing *L. reuteri* (Lr^Reuterin^) or reuterin-nonproducing *L. reuteri* (Lr^No reuterin^) (B) Effect of reuterin on the lifespan of males (left panel) and females (right panel). (**C**) Longevity effects of *L. reuteri* and reuterin under axenic conditions. The closed symbols indicate the survival of conventionally raised flies, and the open symbols indicate the survival of flies under axenic conditions. (**D**) Trans-localization of dFOXO to the nucleus following diet supplementation with reuterin under conventional conditions (upper) or axenic conditions (lower). The negative controls represent the fat body tissue exposed to Cy3-conjugated secondary antibody only (no primary antibody) in addition to DAPI. The abdominal fat body was stained with anti-dFOXO (red) and DAPI (blue). Percentage of the nuclear dFOXO-positive cells compared with the cells stained with DAPI (right, SEM, Conv + control n = 9; Conv + reuterin n = 8; Ax + control n = 9; Ax + reuterin n = 10). Original magnification is 200×. Statistical probability was determined using the *t*-test for Conv flies or Wilcoxon rank sum test for Ax flies. (**E**) The mRNA levels of insulin-like peptide genes and FOXO-target genes were analyzed in male fruit flies fed a reuterin-containing diet or a control diet for five weeks (IIS-related genes, n = 9; FOXO-target genes, n = 15). Statistical probability was determined using the Wilcoxon rank sum test (*dilp1-2*, *dilp4-7*, *thor*, and *inr*) or *t*-test (*dilp3*, *sir2*, and *impl2*). * *p* < 0.05, *** *p* < 0.0001.

The reuterin-producing *L. reuteri* (Lr^reuterin^) and reuterin-nonproducing *L. reuteri* (Lr^No reuterin^) were isolated, and the lifespans of flies under Lr^reuterin^ or Lr^No reuterin^ supplementation were measured to determine if the longevity effect of *L. reuteri* is related to reuterin (Fig. 8A and Supplementary Table 7). Interestingly, the effect of lifespan extension by Lr^reuterin^ supplementation was diminished when the flies were fed Lr^No reuterin^, suggesting that the lifespan extension effect of *L. reuteri* is mediated by reuterin production (Fig. 8A and Supplementary Table 7, male, Lr^No reuterin^, log-rank test, χ^2^ = 0.004, *p* = 0.95). To determine if isolated and purified reuterin can extend the lifespan, the fly lifespan was measured using a concentration range of single-ingredient reuterin from 5 to 500 μg/mL (Fig. 8B and Supplementary Table 8). The lifespan of flies was increased significantly at all concentrations (Fig. 8B and Supplementary Table 8, male, 5 μg/mL, 9% increase, log-rank test, χ^2^ = 4.78, *p* < 0.05; 50 μg/mL, 11% increase, log-rank test, χ^2^ = 7.27, *p* < 0.05; 500 μg/mL, 15% increase, log-rank test, χ^2^ = 12.23, *p* < 0.005). A concentration of 5 μg/mL reuterin was used in subsequent experiments because Chung et al. reported that approximately 32 to 336 μg reuterin was produced per 1 mL of *L. reuteri* [32]. An axenic fly was generated, and the lifespan of flies given 100 μg/mL *L. reuteri* or 5 μg/mL reuterin was measured to determine if the antibacterial effects of reuterin are responsible for the longevity effect. As expected, the longevity effect of *L. reuteri* and reuterin observed in the conventional fly was abolished in the axenic fly (Fig. 8C), suggesting that the longevity effect of *L. reuteri* and reuterin is mediated by the regulation of the bacteria. Supplementation of *L. reuteri* or reuterin decreased the lifespan of flies under axenic conditions (Fig. 8C and Supplementary Table 9, Ax, Lr, 8% decrease, log-rank test, χ^2^ = 13.03, *p* < 0.0005; Reuterin, 5% decrease, log-rank test, χ^2^ = 15.04, *p* < 0.0001). The lifespan of the axenic flies with an antibiotic cocktail was examined to determine how antibiotics change the lifespan of flies under Axenic conditions (Supplementary Fig. 4). Similar to the results of Fig. 8C, the antibiotics cocktail decreased the lifespan of flies under axenic conditions, even though the lifespan was increased by antibiotics under conventional conditions suggesting that the intake of antibiotics adversely affects the longevity under sterile conditions. Furthermore, supplementation of reuterin induced the trans-localization of the dFOXO transcription factor from the cytoplasm to the nucleus in conventional flies (Fig. 8D, *t*-test, *p* < 0.0001), but this trans-localization was diminished significantly in the axenic fly (Fig. 8D, Wilcoxon rank sum test, *p* = 0.06), indicating that activation of the dFOXO transcription factor by reuterin requires the presence of commensal bacteria. The levels of several, but not all *dilp* gene expression, were decreased, and the dFOXO target genes were increased by the administration of reuterin (Fig. 8E, Wilcoxon rank sum test or *t*-test). Overall, the longevity effect of *L*. *reuteri* is mediated by its secondary metabolite, reuterin, probably reducing the bacterial burden of old flies.

## DISCUSSION

Probiotics, which are defined as non-pathogenic commensal bacteria that provide beneficial effects to their host, have been actively studied. These bacteria have anti-inflammatory [10], anti-obesity [33], and anti-diabetic effects [34], but the longevity benefits of probiotic bacteria have not been elucidated clearly. In this study, the data showed that the supplementation of a freeze-dried *L. reuteri* extended the lifespan of *D. melanogaster*, with an optimal concentration of 100 μg/mL. Above 100 μg/mL, *L. reuteri* supplementation did not have any additional lifespan-extension effect like other anti-aging drug candidates, such as rapamycin [35], metformin [36], and Korean red ginseng [37], having an optimal concentration for their longevity effects.

*L. reuteri* supplementation decreased the survival rate of flies under starvation-stress conditions. Consistent with the reduced survival of fruit flies following starvation stress, the body weight and fat contents were also decreased by administering *L. reuteri* without changing the food intake. Similar to the data, many studies have shown that the administration of *L. reuteri* reduces body weight and lipid accumulation in a high-energy-diet rodent model [11-13]. However, several studies using a specific *L. reuteri* strain referred to as *L. reuteri* strain DSM 17938 did not have the anti-obesity effect [11, 13, 38], indicating that *L*. *reuteri* has a strain-dependent anti-obesity effect. This is a human-adapted pro-inflammatory strain that lacks bile salt hydrolase activity [39] and was shown not to alter the body weight and fat content in both diet-induced obese mice [11] and humans [38]. Qiao et al. (2015) also examined the effects of different *L. reuteri* strains on inflammation and fat storage in high-fat diet-induced obese mice. Interestingly, the *L. reuteri* strain isolated from normal mice reduced the body weight, fat content, blood glucose levels, and inflammation, but an *L. reuteri* strain isolated from obese mice failed to reduce the body weight and blood glucose. In the present study, *L. reuteri* ATCC strain SD-5865 was used. Previous studies reported that *L. reuteri* ATCC strain SD-5865 improved insulin resistance [40] and insulin sensitivity in mice and increased insulin secretion in humans [41], indicating that the *L. reuteri* ATCC strain SD-5865 has beneficial effects on the energy metabolism, particularly insulin-related response, in its host. These reports suggest that the studies on the effects of commensal bacteria on the host health should consider the selection of a specific strain as an important factor.

DR, defined as a decrease in nutrient intake without malnutrition, has been well established to improve health and retard the aging process in several organisms, including yeast, nematodes, fruit flies, rodents, and primates [27]. The molecular mechanism for the longevity effect of *L. reuteri* was determined by examining the relationship between DR and *L. reuteri* supplementation and it is shown that the longevity effect of *L. reuteri* supplementation was abolished under DR conditions and was accompanied by the reduction of the IIS pathway. Downregulation of the IIS pathway has been reported to increase the lifespan of many model organisms [42]. FOXO, a potential major mediator in metabolism, cellular proliferation, stress tolerance, and lifespan, is activated by the reduced IIS and is translocated to the nucleus when it is activated. In particular, gene expression of *sir2*, a *Drosophila* homolog of Sirtuin 1, and target genes of FOXO were increased in the flies fed the *L. reuteri* supplement in the present study. Sirtuin 1, a NAD+ dependent deacetylase, was reported to increase the lifespan of diverse organisms, activate the FOXO transcription factor, and act as a mediator of the longevity effect by DR [43]. Furthermore, supplementation with *L. reuteri* delayed the developmental rate of the larva to pupa and larva to adult stages (Fig. 2F). Some studies reported that the reduced IIS pathway is responsible for the delayed developmental rate in fruit fly and DR delayed development in nematodes by reducing the IIS pathway. Thus, the delayed development of the fruit fly fed *L. reuteri* supplement might be due to the inhibited IIS pathway.

Reuterin, an antimicrobial compound produced by *L. reuteri*, is a key metabolite responsible for the longevity effect of *L. reuteri*. Many substances with antimicrobial effects can extend the lifespan of yeast, worms, and fruit flies [44-47]. Recent studies have shown that commensal bacteria are closely related to the lifespan and insulin signaling in the host [26, 48-50]. In particular, controlled bacteria abundance increased the lifespan, and acetic acid-producing *Acetobacter* modulated the IIS pathway in *Drosophila* [26, 49]. Similarly, the life-extension effect of *L. reuteri* and reuterin was related to the reduced abundance of commensal bacteria (Fig. 3 and Supplementary Fig. 3) and the modulation of the IIS pathway in fruit flies (Fig. 5 and Fig. 8). Further studies to examine the relationship between the longevity effect of *L. reuteri* and insulin-regulating bacteria, such as *Acetobacter* will help elucidate the mechanism how the lifespan of the host is extended by the probiotics and commensal bacteria and their causality.

In summary, supplementation with *L. reuteri* and its secondary metabolite, reuterin, can extend the organismal lifespan by inhibiting the IIS pathway and activating the FOXO transcription factor. The probiotic *L. reuteri* can be used as a pro-longevity supplement and a dietary restriction mimetic. This study provides an expanded research field on the effects of probiotics as anti-aging agents.

## Supplementary Materials

The Supplementary data can be found online at: www.aginganddisease.org/EN/10.14336/AD.2023.0122.
