# Supplementary material for: Probiotic Limosilactobacillus Reuteri (Lactobacillus Reuteri) Extends the Lifespan of Drosophila Melanogaster through Insulin/IGF-1 Signaling
Source: Aging Dis. 2023 Aug 1;14(4):1407–24. doi: 10.14336/AD.2023.0122 (PMC10389828; doi:10.14336/AD.2023.0122)
Supplement: Supplementary file 1 — The Supplementary data can be found online at: www.aginganddisease.org/EN/10.14336/AD.2023.0122. [file AD-14-4-1407-s.pdf]

SUPPLEMENTARY DATA

Probiotic *Limosilactobacillus Reuteri* (*Lactobacillus Reuteri*) Extends the Lifespan of *Drosophila Melanogaster* through Insulin/IGF-1 Signaling

Hye-Yeon Lee, Ji-Hyeon Lee, Seung Hyung Kim, Su-Yeon Jo, Kyung-Jin Min

© 2023. Lee HY et al. Published online at http://www.aginganddisease.org/EN/10.14336/AD.2023.0122

SUPPLEMENTARY DATA

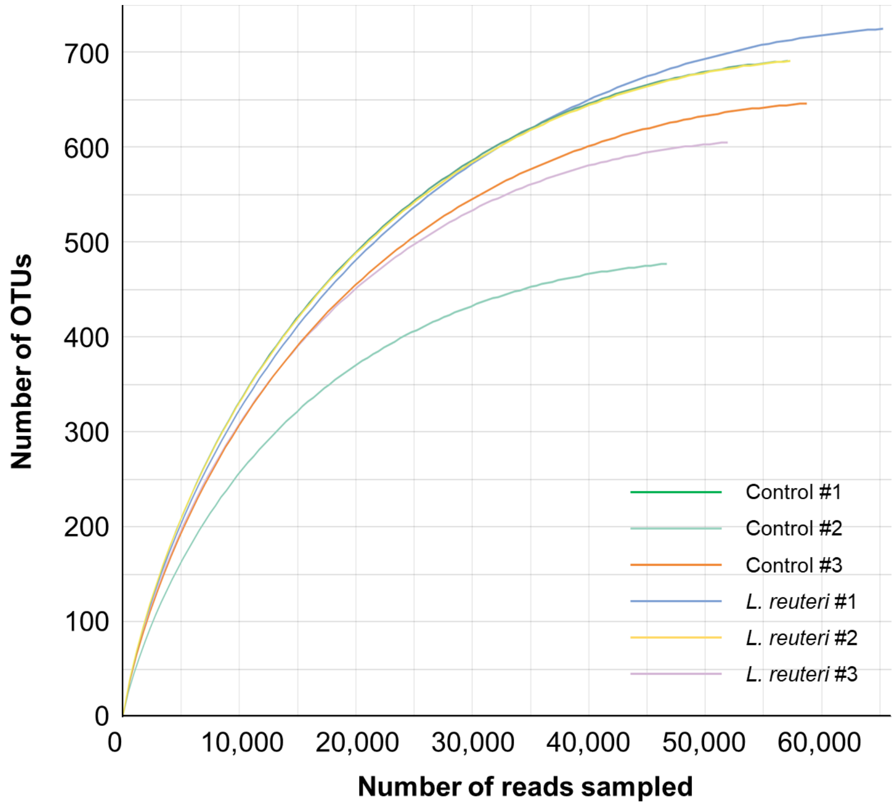

Supplementary Figure 1. Rarefaction curves of operational taxonomic units (OTUs) from Illumina sequencing data. Rarefaction curves of each sample. Rarefaction curves of OTUs clustered at the 97% phylotype similarity level.

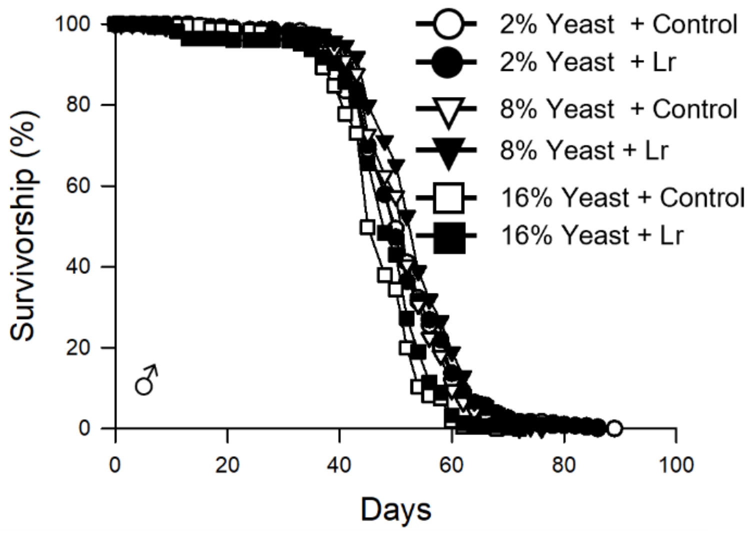

Supplementary Figure 2. Survival of *D. melanogaster* supplemented with *L. reuteri* under 2, 8, or 16% yeast conditions. Survival graph of flies fed 2, 8, or 16% yeast. The open symbols indicate the lifespan of flies fed food without *L. reuteri* (Control) and the closed symbols indicate the lifespan of flies fed food with *L. reuteri* (Lr).

© 2023. Lee HY et al. Published online at http://www.aginganddisease.org/EN/10.14336/AD.2023.0122

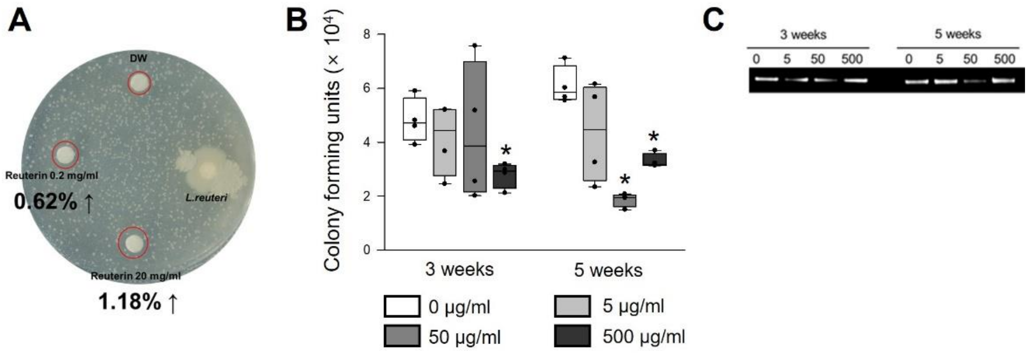

Supplementary Figure 3. Antibacterial effect of reuterin *in vitro* or *in vivo*. (A) Antibacterial activity of reuterin 0.2 mg/ml and 20 mg/ml against bacteria flora from homogenate of fly using disc diffusion test. **(B)** Colony-forming units (CFUs) of fly treated with 5, 50, or 500 µg/mL of reuterin for 3 or 5 weeks. **(C)** PCR assay of microbial 16S rRNA amplified gene using universal (27F, 1492R). Microbial 16S rDNA gene sequences were amplified from genomic DNA extracted from fly treated with 5, 50, or 500 µg/mL of reuterin for 3 or 5 weeks.

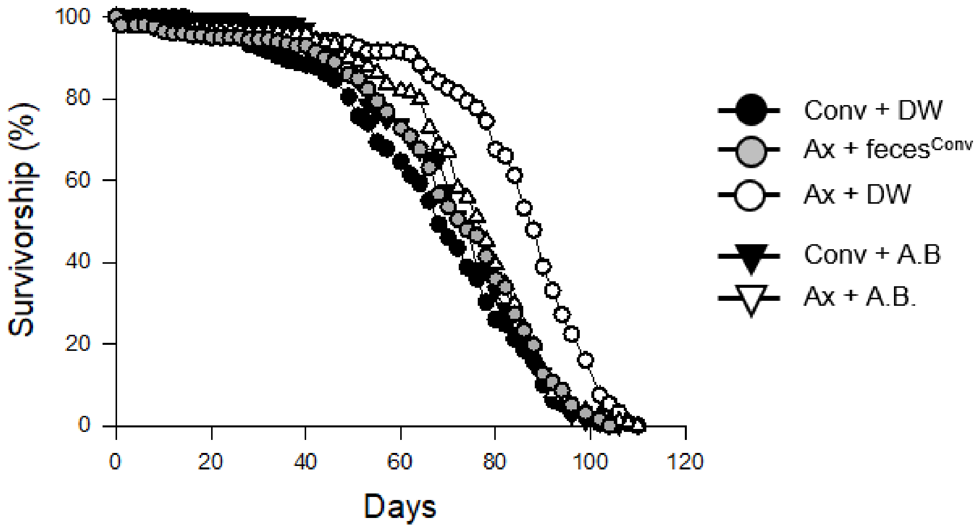

Condition	Treat	n	Mean-lifespan	Median lifespan	Maximum lifespan
Conv	DW	189	66.80 ± 1.48	68	82
	A.B.	186	71.48 ± 1.26	74	84
Ax	feces^Conv	198	70.48 ± 1.54	74	86
	DW	188	83.95 ± 1.39	88	96
	A.B.	188	73.82 ± 1.43	78	86

Supplementary Figure 4. Survival of conventional, feces-fed, antibiotics-fed, and axenic fly. Survival graph (upper) and table (lower) of conventional (black symbols), feces-fed (gray dot), antibiotics-fed (reversed triangle), and axenic (white symbols) fly.

© 2023. Lee HY et al. Published online at http://www.aginganddisease.org/EN/10.14336/AD.2023.0122

SUPPLEMENTARY DATA

Supplementary Table 1. Effect of *Limosilactobacillus reuteri* on lifespan in fruit flies

Sex	Age	Lr (μg/ml)	n	Mean-lifespan	Change (vs. 0 μg/ml)	Median-lifespan	Change (vs. 0 μg/ml)	Maximum-lifespan	Change (vs. 0 μg/ml)	χ^2 (vs. 0 μg/ml)	p-value (vs. 0 μg/ml)	Mortality	R^2
Male	0 day	0	268	58.87 ± 1.31		64		76				y = 0.1191x - 5.9064	0.7684
		50	247	60.51 ± 1.41	3%	62	-3%	81	7%	2.450	0.1139	y = 0.1057x - 5.6794	0.6374
		100	245	67.13 ± 1.34	14%	74	16%	83	9%	27.17	< 0.0001*	y = 0.1254x - 6.4745	0.6908
		250	267	63.91 ± 1.35	9%	67	5%	81	7%	10.12	0.0015*	y = 0.1117x - 5.8998	0.854
Female	0 day	0	265	60.66 ± 1.13		64		74				y = 0.1503x - 6.6262	0.7829
		50	272	66.22 ± 0.95	9%	69	8%	76	3%	15.74	< 0.0001*	y = 0.1492x - 6.8427	0.8542
		100	236	68.85 ±1.05	14%	74	16%	78	5%	42.63	< 0.0001*	y = 0.1496x - 7.0049	0.8063
		250	221	69.20 ±1.13	14%	74	16%	81	9%	48.55	< 0.0001*	y = 0.1462x - 7.0043	0.7939
Male	0 day	0	237	55.85 ± 1.40	8%	62	8%	74	3%	5.32	0.0211	y = 0.0718x - 3.8329	0.2636
		100	247	60.53 ± 1.31		67		76				y = 0.1191x - 5.4836	0.6149
	30 days	0	243	26.36 ± 1.00	17%	20	35%	41	17%	11.92	0.0006*	y = 0.1047x - 3.7723	0.4612
		100	223	30.74 ± 1.23		27		48				y = 0.099x - 3.906	0.5415
Female	0 day	0	250	49.52 ± 1.02	13%	55	9%	62	15%	40.33	< 0.0001*	y = 0.0892x - 3.944	0.338
		100	212	56.00 ± 1.46		60		71				y = 0.0751x - 4.0416	0.2795
	30 days	0	242	39.21 ± 0.81	14%	41	12%	46	20%	40.71	< 0.0001*	y = 0.1523x - 4.4995	0.3952
		100	227	44.6 ± 1.03		46		55				y = 0.1785x - 6.0296	0.807

© 2023. Lee HY et al. Published online at http://www.aginganddisease.org/EN/10.14336/AD.2023.0122

SUPPLEMENTARY DATA

Supplementary Table 2. Richness and diversity estimation of the 16S rRNA gene libraries from the Illumina sequencing analysis

Sample	Target reads	OTUs†	Species richness indices			Species diversity indices		Good's coverage of library (%)
			ACE	CHAO	Jackknife	Shannon	Simpson	
Control #1	57,098	691	715.39	696.93	748	1.43	0.5	99.9
Control #2	46,706	477	498.82	483.41	525	1.38	0.46	99.9
Control #3	58,728	646	665.48	650.31	693	1.43	0.46	99.92
L. reuteri #1	65,278	725	752.85	731.15	785	1.5	0.46	99.91
L. reuteri #2	57,312	691	717.47	696.78	749	1.54	0.44	99.9
L. reuteri #3	51,934	605	624.85	609.66	652	1.53	0.43	99.91

† The operational taxonomic units (OTUs) were defined with pairwise 97% ID.

© 2023. Lee HY et al. Published online at http://www.aginganddisease.org/EN/10.14336/AD.2023.0122

Supplementary Table 3. Commensal bacteria found in the 50 guts of the flies fed *L. reuteri* using Illumina sequencing.

Phylum	Species	Control #1	Control #2	Control #3	*L. reuteri* #1	*L. reuteri* #2	*L. reuteri* #3
Firmicutes	*Lactobacillus sakei* group	69.565	65.458	64.831	65.982	64.367	63.109
Proteobacteria	*Acetobacter persici*	11.228	16.829	19.311	10.806	11.760	12.593
Proteobacteria	*Komagataeibacter medellinensis*	7.598	7.333	5.165	9.316	9.987	10.269
Proteobacteria	*Acetobacter nitrogenifigens* group	4.111	4.661	4.032	6.960	6.911	7.404
Firmicutes	*Lactobacillus*_uc	4.032	2.321	3.693	3.848	4.027	3.764
Proteobacteria	*Acetobacter*_uc	0.849	0.861	1.514	1.353	1.260	1.261
Proteobacteria	*Komagataeibacter*_uc	0.457	0.268	0.298	0.591	0.623	0.585
Proteobacteria	Variovorax paradoxus group	0.800	0.662	0.209	0.173	0.265	0.177
Proteobacteria	*Phyllobacterium myrsinacearum* group	0.112	0.362	0.027	0.034	0.030	0.027
Bacteroidetes	*Bacteroides*_uc	0.072	0.066	0.058	0.041	0.023	0.010
Proteobacteria	*Pelomonas saccharophila* group	0.044	0.034	0.039	0.026	0.007	0.008
Actinobacteria	*Bifidobacterium pseudolongum* group	0.042	0.039	0.017	0.014	0.023	0.019
Firmicutes	GG697149_s group	0.037	0.019	0.015	0.011	0.014	0.008
Firmicutes	*Blautia wexlerae*	0.030	0.024	0.027	0.014	0.005	0.002
Actinobacteria	*Bifidobacterium gallinarum* group	0.018	0.019	0.014	0.014	0.009	0.012
Firmicutes	*Anaerostipes hadrus* group	0.012	0.036	0.015	0.009	0.005	0.004
Proteobacteria	*Acetobacter aceti* group	0.009	0.015	0.012	0.020	0.016	0.006
Proteobacteria	*Bradyrhizobium japonicum* group	0.018	0.017	0.017	0.008	0.007	0.008
Firmicutes	*Megamonas rupellensis* group	0.021	0.004	0.009	0.011	0.005	0.012
Firmicutes	LN913006_s group	0.011	0.017	0.010	0.014	0.007	0.002
Firmicutes	*Roseburia cecicola* group	0.014	0.021	0.009	0.006	0.005	0.002
Actinobacteria	*Collinsella aerofaciens* group	0.009	0.013	0.015	0.003	0.009	0.008
Firmicutes	*Lactobacillus reuteri* group	0.002	0.004	0.000	0.017	0.014	0.019
Bacteroidetes	*Prevotella*_uc	0.012	0.011	0.002	0.003	0.005	0.006
Bacteroidetes	*Bacteroides ovatus* group	0.016	0.006	0.009	0.008	0.000	0.000
Bacteroidetes	*Bacteroides acidifaciens* group	0.012	0.009	0.005	0.005	0.004	0.004
Firmicutes	*Subdoligranulum variabile* group	0.005	0.009	0.007	0.005	0.007	0.000
Firmicutes	*Fusicatenibacter saccharivorans*	0.007	0.011	0.003	0.005	0.002	0.002
Firmicutes	*Faecalibaculum rodentium*	0.005	0.006	0.005	0.005	0.002	0.002
Firmicutes	*Dorea longicatena*	0.007	0.006	0.002	0.008	0.002	0.000
Bacteroidetes	*Bacteroides coprocola*	0.007	0.002	0.003	0.005	0.004	0.002
Firmicutes	*Ruminococcus faecis*	0.004	0.006	0.007	0.003	0.002	0.000
Proteobacteria	*Sutterella stercoricanis* group	0.002	0.011	0.000	0.002	0.002	0.004
Proteobacteria	*Sutterella wadsworthensis*	0.009	0.009	0.002	0.000	0.000	0.000
Bacteroidetes	Bacteroides thetaiotaomicron group	0.002	0.004	0.005	0.002	0.000	0.006
Bacteroidetes	*Bacteroides stercoris*	0.005	0.009	0.002	0.002	0.000	0.000
Proteobacteria	*Ralstonia pickettii* group	0.005	0.009	0.002	0.002	0.000	0.000
Firmicutes	*Ruminococcus bromii* group	0.002	0.006	0.003	0.000	0.004	0.002
Proteobacteria	*Sphingomonas trueperi* group	0.002	0.006	0.005	0.000	0.002	0.002
Firmicutes	*Veillonella dispar*	0.005	0.004	0.002	0.000	0.000	0.004
Proteobacteria	*Haemophilus parainfluenzae* group	0.004	0.000	0.003	0.003	0.005	0.000
Firmicutes	*Coprococcus comes*	0.007	0.004	0.002	0.000	0.000	0.002
Firmicutes	*Clostridium celatum* group	0.004	0.004	0.005	0.002	0.000	0.000
Firmicutes	*Streptococcus salivarius* group	0.004	0.002	0.000	0.003	0.002	0.004
Firmicutes	*Faecalibacterium prausnitzii* group	0.002	0.011	0.002	0.000	0.000	0.000
Actinobacteria	*Bifidobacterium bifidum*	0.004	0.004	0.002	0.000	0.000	0.002
Firmicutes	*Megasphaera elsdenii*	0.005	0.002	0.003	0.000	0.000	0.000
Firmicutes	*Bacillus megaterium* group	0.002	0.000	0.000	0.002	0.004	0.004
Firmicutes	*Intestinibacter bartlettii*	0.004	0.004	0.002	0.000	0.000	0.000
Firmicutes	*Romboutsia timonensis* group	0.002	0.002	0.005	0.000	0.000	0.000
Proteobacteria	*Acetobacter lovaniensis* group	0.000	0.000	0.002	0.002	0.004	0.002

© 2023. Lee HY et al. Published online at http://www.aginganddisease.org/EN/10.14336/AD.2023.0122

SUPPLEMENTARY DATA

Supplementary Table 4. Lifespan of *D. melanogaster* supplemented with *L. reuteri* under 2, 8, or 16% yeast conditions.

Sex	Group		n	Mean-lifespan	Change	Median-lifespan	Change	Maximum-lifespan	Change	χ^2	p-value
Male	Control	2%	297	51.23 ± 0.56	9%	50	11%	58	12%	41.91	< 0.0001*
		8%	294	51.11 ± 0.54	9%	52	16%	56	8%	46.94	< 0.0001*
		16%	271	46.83 ± 0.49		45		52			
	Lr	2%	264	51.36 ± 0.60	6%	50	4%	58	7%	17.10	< 0.0001*
		8%	285	53.35 ± 0.54	11%	54	13%	60	11%	53.96	0.0001*
		16%	279	48.23 ± 0.58		48		54			
Female	Control	2%	216	72.5 ± 1.11	25%	76	27%	82	17%	159.52	< 0.0001*
		8%	245	71.72 ± 0.79	24%	72	20%	80	14%	150.43	< 0.0001*
		16%	276	57.88 ± 0.85		60		70			
	Lr	2%	281	74.70 ± 0.76	19%	78	22%	82	11%	188.84	< 0.0001*
		8%	287	79.53 ± 0.64	27%	82	28%	86	16%	318.43	< 0.0001*
		16%	283	62.74 ± 0.71		64		74			
Male	2%	Control	297	51.23 ± 0.56	0%	50	0%	58	0%	0.03	0.8666
		Lr	264	51.36 ± 0.60		50		58			
	8%	Control	294	51.11 ± 0.54	4%	52	4%	56	7%	10.93	0.0009*
		Lr	285	53.35 ± 0.54		54		60			
	16%	Control	271	46.83 ± 0.49	3%	45	7%	52	4%	8.09	0.0044*
		Lr	279	48.23 ± 0.58		48		54			
Female	2%	Control	216	72.5 ± 1.11	3%	76	3%	82	0%	0.35	0.5557
		Lr	281	74.7 ± 0.76		78		82			
	8%	Control	245	71.72 ± 0.79	11%	72	14%	80	8%	69.44	< 0.0001*
		Lr	287	79.53 ± 0.64		82		86			
	16%	Control	276	57.88 ± 0.85	8%	60	7%	70	6%	16.85	< 0.0001*
		Lr	283	62.74 ± 0.71		64		74			

© 2023. Lee HY et al. Published online at http://www.aginganddisease.org/EN/10.14336/AD.2023.0122

SUPPLEMENTARY DATA

Supplementary Table 5. Lifespan of mutant fly with *L. reuteri* supplementation

Group		n	Mean-lifespan	Change	Median-lifespan	Change	Maximum-lifespan	Change	χ^2	p-value
w^{1118}	Control	270	69.45 ± 0.93	5%	75	0%	82	0%	12.34	0.0004*
	Lr	284	72.99 ± 0.90		75		82			
$chcio^{1/+}$	Control	275	59.97 ± 0.96	3%	63	0%	70	3%	1.27	0.2594
	Lr	289	61.96 ± 0.85		63		72			
$sir2^{2A\text{-}7\text{-}11}$	Control	271	55.01 ± 0.84	-8%	56	-4%	65	-6%	10.36	0.0013*
	Lr	239	50.75 ± 0.95		54		61			
yw	Control	227	43.40 ± 0.67	4.54	45	4.44	49	6.12	9.65	0.0019*
	Lr	266	45.36 ± 0.65		47		52			
$foxo^{21/+}$	Control	250	43.86 ± 0.98	-1.79	47	4.26	54	3.70	2.42	0.1201
	Lr	310	43.08 ± 1.08		49		56			
$foxo^{25/+}$	Control	256	53.68 ± 1.01	-1.78	59	-5.08	67	-5.97	1.82	0.1768
	Lr	245	52.72 ± 0.95		56		63			
$foxo^{21/25}$	Control	270	38.47 ± 1.04	1.13	40	0.00	49	6.12	0.17	0.6834
	Lr	248	38.90 ± 1.12		40		52			
Da-gal4	Control	261	53.50 ± 0.78	7%	57	4%	61	3%	27.33	< 0.0001*
	Lr	260	57.46 ± 0.66		59		63			
Da-gal4 > UAS-AMPK	Control	299	73.93 ± 0.86	-1%	75	0%	79	0%	1.17	0.2791
	Lr	292	73.29 ± 0.60		75		79			
Da-gal4 > UAS-$S6K^{KQ}$	Control	287	61.27 ± 0.66	-1%	63	0%	67	-3%	0.35	0.5548
	Lr	263	60.48 ± 0.71		63		65			

© 2023. Lee HY et al. Published online at http://www.aginganddisease.org/EN/10.14336/AD.2023.0122

SUPPLEMENTARY DATA

Supplementary Table 6. Serum parameters after feeding experimental diets in mice.

	WT	HFD	HFD + Lr	p-value		
				WT vs. HFD	WT vs. HFD + Lr	HFD vs. HFD + Lr
Creatine (mg/dL)	0.5 ± 0.01	0.5 ± 0.01	0.5 ± 0.01	0.3579	0.0413*	0.1339
Glucose (mg/dL)	135.1 ± 7.0	228.9 ± 10.83	186.1 ± 13.5	< 0.0001*	0.0057*	0.0230*
AST (U/L)	94.2 ± 7.7	129.3 ± 12.6	94.4 ± 5.4	0.0287*	0.9799	0.0260*
ALT (U/L)	28.2 ± 1.2	70.5 ± 8.12	32.1 ± 2.2	0.0006*	0.1300	0.0010*
Triglyceride (mg/dL)	99.4 ± 5.2	124.5 ± 6.5	120.6 ± 5.0	0.0071*	0.0094*	0.6406
Total cholesterol (mg/dL)	119.5 ± 3.1	231.8 ± 11.5	185.3 ± 8.9	< 0.0001*	< 0.0001*	0.0058*
HDL cholesterol (mg/dL)	86.9 ± 2.8	102 ± 3.3	102.3 ± 4.1	0.0029*	0.0058*	0.9499
LDL cholesterol (mg/dL)	11.4 ± 0.6	31.2 ± 2.4	21.9 ± 1.0	< 0.0001*	< 0.0001*	0.0037*
Free fatty acid (mM/dL)	3.7 ± 0.1	4.41 ± 0.1	4.08 ± 0.1	< 0.0001*	0.0084*	0.0163*
Adiponectin (ng/ml)	806.8 ± 61.5	398.4 ± 44.6	717.4 ± 33.8	0.0017*	0.2499	0.0013*
Leptin (ng/ml)	0.7 ± 0.1	1886.6 ± 210.3	142.2 ± 81.7	0.0029*	0.1816	0.0002*
IGF-1 (ng/ml)	8.8 ± 0.6	14.5 ± 1.5	7.6 ± 1.8	0.0134*	0.5627	0.0267*

Values are presented as mean ± SEM. n=5-10. AST, aspartate aminotransferase; ALT, alanine aminotransferase; IGF-1, insulin-like growth factor-1.

© 2023. Lee HY et al. Published online at http://www.aginganddisease.org/EN/10.14336/AD.2023.0122

SUPPLEMENTARY DATA

Supplementary Table 7. Lifespan of *D. melanogaster* supplemented with reuterin-producing *L. reuteri*

Sex	Treat	n	Mean-lifespan	Change (vs. 0 µg/ml)	Median-lifespan	Change (vs. 0 µg/ml)	Maximum-lifespan	Change (vs. 0 µg/ml)	χ^2 (vs. 0 µg/ml)	p-value (vs. 0 µg/ml)	Mortality	R^2
Male	Control	285	39.72 ± 1.04		41		57				y = 0.1139x - 4.7458	0.5974
	$Lr^{reuterin}$	324	42.32 ± 0.99	7%	48	17%	59	4%	5.9674	0.0146*	y = 0.1515x - 5.2582	0.7106
	$Lr^{no\ reuterin}$	297	41.40 ± 0.95	4%	48	17%	55	-4%	0.0043	0.9475	y = 0.1563x - 5.2665	0.7694
Female	Control	270	40.80 ± 0.62		43		48				y = 0.2525x - 6.7036	0.9021
	$Lr^{reuterin}$	282	44.97 ± 0.59	10%	48	12%	50	4%	45.3965	< 0.0001*	y = 0.2458x - 6.9116	0.6809
	$Lr^{no\ reuterin}$	284	40.77 ±0.61	0%	43	0%	48	0%	0.005	0.9435	y = 0.2681x - 6.9898	0.8386

© 2023. Lee HY et al. Published online at http://www.aginganddisease.org/EN/10.14336/AD.2023.0122

SUPPLEMENTARY DATA

Supplementary Table 8. Lifespan of *D. melanogaster* with reuterin.

Sex	Reuterin (μg/ml)	n	Mean-lifespan	Change (vs. 0 μg/ml)	Median-lifespan	Change (vs. 0 μg/ml)	Maximum-lifespan	Change (vs. 0 μg/ml)	χ^2 (vs. 0 μg/ml)	p-value (vs. 0 μg/ml)	Mortality	R^2
Male	0	283	44.45 ± 1.26		43		61				y = 0.0373x - 4.4371	0.6306
	5	254	48.34 ± 1.38	9%	46	7%	69	13%	4.78	0.0288*	y = 0.0413x - 4.8838	0.5859
	50	228	49.29 ± 1.52	11%	46	7%	71	16%	7.27	0.007*	y = 0.0422x - 4.914	0.5634
	500	241	50.90 ± 1.47	15%	46	7%	71	16%	12.23	0.0005*	y = 0.0418x - 4.9763	0.614
Female	0	264	44.86 ± 0.76		48		53				y = 0.0861x - 6.0944	0.78
	5	240	47.60 ± 0.82	6%	50	4%	56	6%	9.67	0.0019*	y = 0.0876x - 6.3593	0.8545
	50	242	46.63 ± 0.81	4%	50	4%	53	0%	3.79	0.0515	y = 0.0822x - 6.2639	0.7959
	500	248	47.62 ± 0.85	6%	50	4%	56	6%	13.70	0.0002*	y = 0.0809x - 6.2426	0.825

© 2023. Lee HY et al. Published online at http://www.aginganddisease.org/EN/10.14336/AD.2023.0122

SUPPLEMENTARY DATA

Supplementary Table 9. Effect of *L. reuteri* and reuterin on lifespan in Ax fruit flies.

Group		n	Mean-lifespan	Change (vs. Control)	Median-lifespan	Change (vs. Control)	Maximum-lifespan	Change (vs. Control)	χ^2 (vs. Control)	p-value (vs. Control)
Conv.	Control	199	42.62 ± 0.81		42		51			
	Lr	194	45.27 ± 0.84	6%	46.5	11%	51	0%	7.9579	0.0048*
	Reuterin	189	45.11 ± 0.82	6%	48	14%	51	0%	5.2423	0.0220*
Ax	Control	192	52.22 ± 0.69		54		57			
	Lr	196	48.20 ± 0.75	-8%	49.5	-8%	54	-5%	13.0311	0.0003*
	Reuterin	179	49.51 ± 0.66	-5%	51	-6%	54	-5%	15.0434	0.0001*

© 2023. Lee HY et al. Published online at http://www.aginganddisease.org/EN/10.14336/AD.2023.0122

Supplementary Table 10. Composition of fly husbandry food and bacteria incubation media.

Food	Composition
Cornmeal-sugar-yeast (CSY) media	5.2% cornmeal
	11% sugar
	2.6% instant yeast
	0.5% propionic acid
	0.04% methyl 4 hydroxybenzoate (Sigma Aldrich, MO, USA)
	0.8% (for Conv food) or 1% agar (for Ax food)
Sugar-yeast (SY) media	10% sugar
	10% yeast
	0.8% agar
	0.5% propionic acid
	0.04% methyl 4 hydroxybenzoate (Sigma Aldrich, MO, USA)
Cornmeal-sugar-yeast (CSY) media for DR	5.2% cornmeal
	11% sugar
	2, 8, or 16% brewer's yeast
	0.8% agar
	0.5% propionic acid
	0.04% methyl 4 hydroxybenzoate (Sigma Aldrich, MO, USA)
Plate count agar (PCA)	0.5% tryptone
	0.25% yeast extract
	0.1% glucose
	1.5% bacto agar
Lactobacillus-selective (MRS) media	1% peptone
	1% beef extract
	0.5% yeast extract
	2% dextrose
	0.1% polysorbate
	0.2% ammonium citrate
	0.5% sodium acetate
	0.01% magnesium sulfate
	0.005% manganese sulfate
	0.2% dipotassium phosphate
Acetobacter-selective media	2.5% D-mannitol (BD & Difco)
	0.5% yeast extract (BD & Difco)
	0.3% peptone (BD & Difco)
	1.5% bacto agar

© 2023. Lee HY et al. Published online at http://www.aginganddisease.org/EN/10.14336/AD.2023.0122

SUPPLEMENTARY DATA

Supplementary Table 11. Sequences of primers for PCR.

Primer	Forward (5'-3')	Reverse (5'-3')
Rp49	ATC GGT TAC GGA TCG AAC AA	GAC AAT CTC CTT GCG CTT CT
Dilp1	AAT GGC AAT GGT CAC GCC GAC TGG	GCT GTT GCC CAG CAA GCT TTC ACG
Dilp2	ACG AGG TGC TGA GTA TGG TGT GCG	CAC TTC GCA GCG GTT CCG ATA TCG
Dilp3	GTC CAG GCC ACC ATG AAG TTG TGC	CTT TCC AGC AGG GAA CGG TCT TCG
Dilp4	TGG ATT TAC ACG CCG TGT CAG GCG	ACA CCC TTC TCC GTA TCC GCA TGG
Dilp5	TGT TCG CCA AAC GAG GCA CCT TGG	CAC GAT TTG CGG CAA CAG GAG TCG
Dilp6	TGC TAG TCC TGG CCA CCT TGT TCG	GGA AAT ACA TCG CCA AGG GCC ACC
Dilp7	GAG CTG TAC TCC TGT TCG TCC TGC	TCC AAG CCT CAT CAT TGC CCG TCC
Sir2	CAC GAC CGT TCT ACA AGT TT	GCA GCT CCT CCT CAG TAA C
Thor	GAA GGT TGT CAT CTC GGA TCC	ATG AAA GCC CGC TCG TAG
ImpL2	GCC GAT ACC TTC GTG TAT CC	TTT CCG TCG TCA ATC CAA TAG
InR	TAC TCG GAG CAT TGG AGG CAT	AAC AGT GGC GGA TTC GGT T
Universal primer (27F, 1492R)	AGA GTT TGA TCM TGG CTC AG	TAC GGY TAC CTT GTT ACG ACT T
Lactobacillus-selective primer	GCA AGG CTG AAA CTC AAA GG	TTC ATG TAG GCG AGT TGC AG
Acetobacter-selective primer	CCC TTA TGT CCT GGG CTA CA	TAC GGY TAC CTT GTT ACG ACT T

© 2023. Lee HY et al. Published online at http://www.aginganddisease.org/EN/10.14336/AD.2023.0122
